# Capture-C: A modular and flexible approach for high-resolution chromosome conformation capture

**DOI:** 10.1038/s41596-021-00651-w

**Published:** 2022-02-04

**Authors:** Damien J. Downes, Alastair L. Smith, Magdalena A. Karpinska, Taras Velychko, Kevin Rue-Albrecht, David Sims, Thomas A. Milne, James O.J. Davies, A. Marieke Oudelaar, Jim R. Hughes

**Affiliations:** 1 MRC Molecular Haematology Unit, MRC Weatherall Institute of Molecular Medicine, University of Oxford, Oxford, UK; 2 MRC WIMM Centre for Computational Biology, MRC Weatherall Institute of Molecular Medicine, University of Oxford, Oxford, UK; 3 Max Planck Institute for Biophysical Chemistry, Göttingen, Germany; 4 Department of Molecular Biology, Max Planck Institute for Biophysical Chemistry, Göttingen, Germany; 5 NIHR Oxford Biomedical Research Centre, Haematology Theme, Oxford, UK

**Keywords:** Chromosome conformation capture, Chromatin structure, Gene regulation, Nuclear organization

## Abstract

Chromosome conformation capture (3C) methods measure the spatial proximity between DNA elements in the cell nucleus. Many methods have been developed to sample 3C material, including the Capture-C family of protocols. Capture-C methods use oligonucleotides to enrich for interactions of interest from sequencing-ready 3C libraries. This approach is modular and has been adapted and optimised to work for sampling of disperse DNA elements (NuTi Capture-C), including from low-cell inputs (LI Capture-C), as well as to generate Hi-C like maps for specific regions of interest (Tiled-C) and to interrogate multi-way interactions (Tri-C). We present the design, experimental protocol and analysis pipeline for NuTi Capture-C in addition to the variations for generation of LI Capture-C, Tiled-C and Tri-C data. The entire procedure can be performed in three weeks and requires standard molecular biology skills and equipment, access to a next-generation sequencing platform, and basic bioinformatic skills. Implemented with other sequencing technologies, these methods can be used to identify regulatory interactions and to compare the structural organisation of the genome in different cell types and genetic models.

## Introduction

### Development of Capture-C methods

Chromosome conformation capture (3C) is a powerful method to measure the proximity of DNA elements within the three-dimensional confines of the nucleus^1^. All 3C methods follow a general principle of chromatin digestion and re-ligation, with minimal disruption of nuclear structure achieved either by fixation or careful buffering to maintain native conditions^2^. Chimeric ligation junctions are then assayed, with more frequent ligation between two distal fragments being a proxy for greater proximity. Originally, chimeric junctions were assayed directly in a low throughput manner using PCR with specifically targeted primer pairs^1^. The application of next-generation sequencing to assay ligation junctions has allowed high-throughput sampling of interactions in all-versus-all approaches, most commonly *in situ* Hi-C at relatively low resolution^3^, and many-versus-all approaches at high resolution, commonly with the Capture-C^4,5^ or 4C-seq^6^ methods.

**Figure 1:**
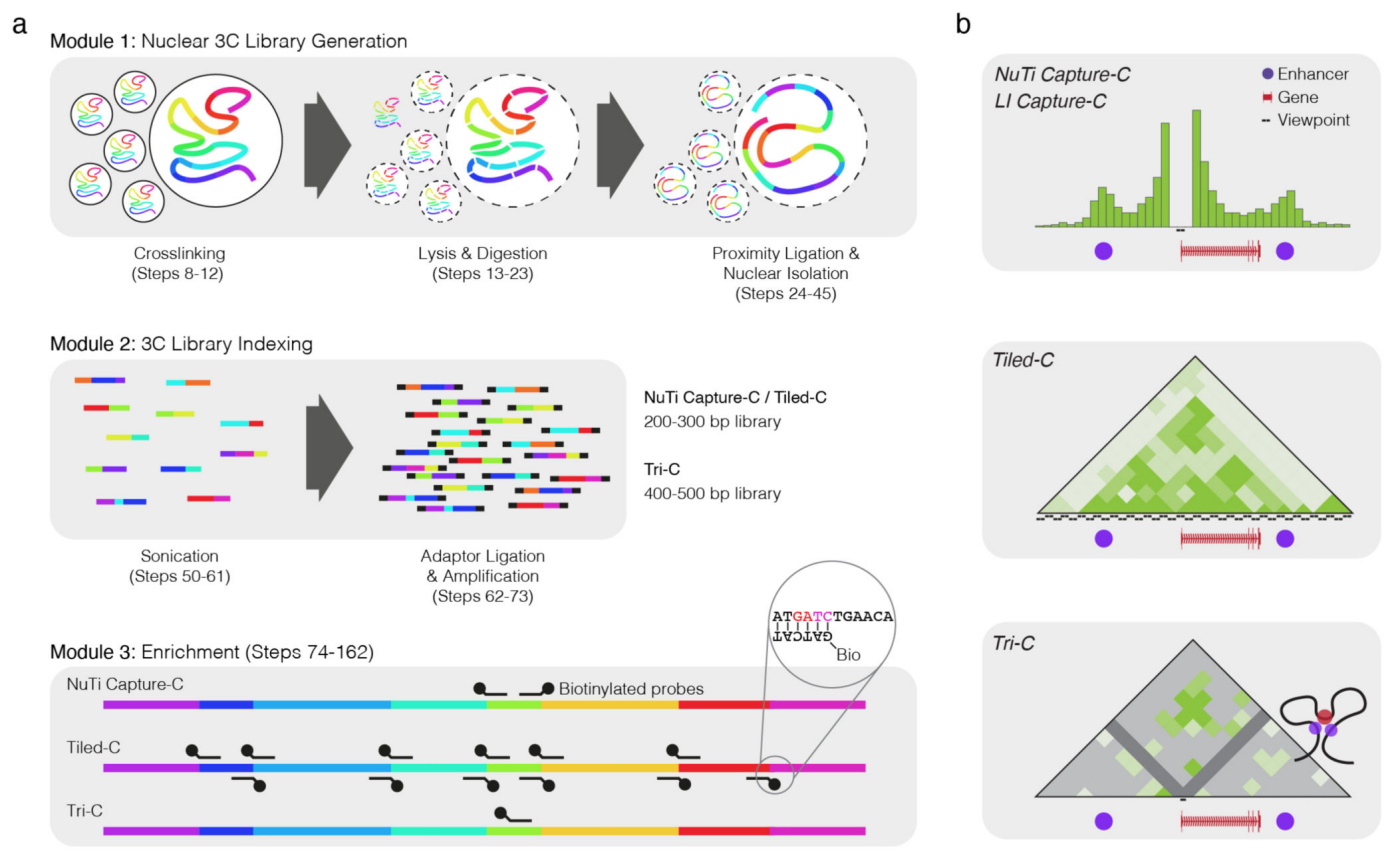
Capture-C is modular and adaptable for characterizing chromatin folding. **a,** The Capture-C family of methods involves three distinct modules. In the first module a Nuclear 3C library is generated from 2% formaldehyde fixed cells that are lysed, then permeabilised with SDS, and digested with a frequent 4-base cutter (*DpnII* or *Nla*III). Proximity ligation re-arranges the genome order to reflect spatial 3D organisation. Finally, for this module, centrifugation is used to separate DNA from ruptured nuclei from DNA in intact nuclei, which contains more informative 3C material. Library indexing in module 2 is performed using standard next-generation sequencing kits with sonication providing unique ends for PCR duplicate filtering. For Tri-C, gentler sonication is used to generate longer fragments which contain multiple ligation junctions. The third module is the most diverse, with a unique oligonucleotide design for each method. NuTi Capture-C uses a pair of oligonucleotides from the same strand of DNA that overlap restriction digestion sites of disperse fragments. For Tiled-C the same approach is used, however contiguous fragments are targeted and double stranded oligonucleotides have typically been used. In Tri-C a single oligonucleotide in the centre of a short restriction fragments enriches for sonication fragments with multiple ligation junctions. **b**, Schematic of results for a hypothetical locus, with one gene (red) and two enhancers (purple circles). NuTi Capture-C, or the low-cell variation LI Capture-C, from the promoter can be used to show direct interactions with both enhancers, Tiled-C produces a Hi-C like interaction map showing the three elements are in a TAD-like regulatory domain, and Tri-C shows that the two enhancers can be found simultaneously interacting with each other and the promoter at single alleles.

The first Capture-C^4^ method was established as a many-versus-all approach that used RNA oligonucleotide pull-down of restriction fragments of interest from *in situ* 3C material. Subsequent sequencing allowed detection of interacting fragments in an unbiased manner. This approach was later applied to Hi-C libraries to develop Capture Hi-C (CHi-C), often called Promoter Capture Hi-C^7^, and most recently dubbed Enhancer Capture Hi-C^8^. Capture-C was improved by the application of biotinylated single stranded DNA (ssDNA) oligonucleotides for sequential “double capture” of indexed and multiplexed 3C libraries^5^. This improved method, Next Generation (NG) Capture-C, achieved 30-50% on-target sequencing efficiency, with 10,000-100,000+ unique reporters per viewpoint. Most-importantly, as 3C libraries used in Capture-C methods are indexed after sonication, PCR duplicates can be distinguished and excluded from analysis. The Capture-C approach can be divided into three distinct modules: 3C library generation, indexing, and enrichment (Fig. 1a). By careful optimisation of the library generation and indexing steps, the cell requirement was reduced from >1M cells to as few as 10,000 cells for Low-Input Capture-C (LI Capture-C)^9^. Subsequent work to reduce protocol inefficiencies from the *in situ* 3C library generation module led to the development of Nuclear 3C, whereby intact nuclei are recovered after ligation, reducing the frequency of spurious ligation events between nuclei 3.3-fold^10^. Similarly, optimisation of the enrichment step established Titrated Capture-C, which can achieve 30-50% on targeting sequencing efficiency after a single capture enrichment step^10^. The combination of Nuclear 3C libraries, the indexing efficiency improvements of LI Capture-C, and the additive effects of Titrated Capture-C and double capture from NG Capture-C into a single method, Nuclear Titrated (NuTi) Capture-C, provides a highly sensitive approach for many-versus-all 3C experiments. NuTi Capture-C provides as high as 98% on target sequencing and has been used to interrogate the regulatory interactions of 8,000 erythroid promoters simultaneously^10^.

In addition to allowing systematic optimisation, the modular nature of the Capture-C method has enabled development of techniques for asking diverse and nuanced questions (Fig. 1b). By altering the size of fragments generated at the sonication step of indexing, Tri-C allows the interrogation of multi-way interactions for inference of higher-order configurations and structures^11,12^. Similarly, using oligonucleotides targeting contiguous fragments in Mb-sized regions (rather than disperse fragments), allows Tiled-C to generate high-resolution many-versus-many contact matrices akin to Hi-C^13^. Tiled-C was also the first Capture-C method to implement double standed DNA (dsDNA) oligonucleotides, though PCR generated dsDNA oligonucleotides have also been used to target dispersed elements^14^. Because of the inherent efficiency, Tiled-C can generate high-quality data in only a few thousand cells. The matrices generated with Tiled-C provide the ability to analyse topologically associated domains (TADs), without compromising on the high-resolution details provided by the other Capture-C methods.

### Applications of Capture-C methods

Capture-C methods can be applied to analyse any fragment or region of the genome which is amenable to probe design. Therefore, Capture-C methods have been applied to numerous biological questions across a diverse range of organisms and species including human, mouse, fly, and chicken. This has allowed for exploration of the roles of enhancers, super-enhancers and promoter competition in the regulation of gene expression in numerous contexts^10,13,15–23^. Similarly, insights into the dynamics of polycomb bodies, X-chromosome inactivation and CTCF boundaries have been achieved by targeting appropriate elements in a range of genetic models^24–31^. Capture-C has also been applied to understand human disease, through mapping the interactions of regulatory polymorphisms associated with complex traits^32–34^, and to help determine the effects of monogenic disease-causing mutations^35–38^. Capture-C methods have also been applied in conjunction with other methods; to validate TADs predicted by the DeepC machine learning algorithm^39^, to complement findings from high-resolution RASER-FISH^40^, and as input for and validation of polymer models of higher-order chromatin structure^41^.

### Comparison with other methods

#### Disperse viewpoint targeting (many-versus-all)

NuTi Capture-C is primarily applied in many-versus-all experiments to determine interactions for genomic elements of interest. Numerous approaches have been developed for this type of experiment. Early sequencing 3C methods, including the original Capture-C^4^ and 4C-seq^42,43^, provided low depth of signal or lacked the ability to distinguish PCR duplicates. For this reason, some researchers have preferred 3C-qPCR^44^, which was thought to be more quantitative, but it does not achieve many-versus-all data as primers need to be designed and optimised for all fragment pairs of interest, resulting in extremely low-resolution profiles. Improvements to the sequencing-based 3C approaches, NG Capture-C^5^ and UMI-4C^45^, provided greater depth of signal and allowed PCR duplicates to be filtered by use of unique sonication ends (this was possible with the original Capture-C as well), overcoming previous limitations and allowing high-throughput analysis at tens to hundreds of targets; with NG Capture-C providing the greater number of unique reporters per viewpoint^46^. The application of many-versus-all experiments to thousands of targets was initially limited to CHi-C^7,47^. By performing pull-down in Hi-C libraries, 3C material is enriched for successful ligation junctions, at the expense of library complexity due to the relative inefficiency of the molecular steps required to generate Hi-C libraries. Excluding these inefficient steps allows NuTi Capture-C to generate up to 1,000-fold greater depth of signal than Capture Hi-C^46^. CHi-C experiments, generally target in the region of 20,000 promoters or enhancers, but using infrequent-cutting enzymes in few replicates that result in low-resolution data (1-10 kb resolution with 100-1,000 interactions per viewpoint^46^). However, with careful design and optimisation, NuTi Capture-C has been applied to ~8,000 active erythroid promoters at high-resolution in triplicate^10^, indicating that genome-scale experiments are no longer limited to lower-resolution approaches.

3C resolution can be increased by using deoxyribonuclease (DNase I) or micrococcal nuclease (MNase) to digest chromatin^48–50^ instead of restriction endonucleases, as these enzymes have no specific cutting motif. MNase-digested 3C libraries were initially used in all-versus-all approaches^48^. Recently, we have reported a new approach in which MNase digestion is combined with a targeted enrichment method, similar to NuTi Capture-C. Micro Capture-C (MCC) provides super-high-resolution 3C data for selected viewpoints, and even permits the footprinting of transcription factor binding at promoters and enhancers^51^. Careful optimisation of micrococcal nuclease levels is needed to achieve super-high-resolution data. Therefore, MCC requires tens of millions of cells and is currently not easily applied to low-abundance primary cell populations, in contrast to traditional Capture-C methods, though this will undoubtedly change as the MCC protocol is refined and optimised.

The Capture-C, Capture Hi-C, and MCC methods use defined sequence specific oligonucleotides for enrichment. Other many-versus-all approaches use 3C combined with immunoprecipitation of proteins (ChIA-PET^52^, PLAC-seq^53^, ChIA-DROP^54^, Hi-ChIP^55^) or RNA (Hi-ChIRP^56^) to achieve enrichment. These methods enticingly allow the simultaneous identification of protein binding sites or enhancers and their interactions. In reality the results are difficult to interpret because they are prone to bias caused by enrichment. This means they generally over report that sites enriched for the targeted molecule contact other similarly enriched sites. Mathematical and experimental quantification of the bias induced between two simultaneously enriched distant sites (i.e. co-targeting) shows that it is incredibly difficult to accurately correct^10^, as such, no method is generally applied in these hybrid technologies. In comparison, the defined nature of oligonucleotide pull-down in Capture-C, Capture Hi-C and MCC experiments allows the exclusion of biased fragments from analyses, providing more robust and interpretable findings.

#### Contiguous viewpoint targeting (many-versus-many)

Tiled-C was designed to combine the ability of all-versus-all 3C methods such as Hi-C to map large-scale chromatin structures including TADs, and the ability of one/many-versus-all 3C methods such as NuTi Capture-C to identify enhancer-promoter interactions within TADs at high resolution. While NuTi Capture-C targets disperse individual restriction fragments as viewpoints, Tiled-C uses a panel of capture oligonucleotides tiled across all contiguous restriction fragments within specified genomic regions. This allows for efficient enrichment for interactions within this region and thus for deep, targeted sequencing of these chromatin interactions. Although co-targeting of distal fragments induces enrichment bias^10^, the contiguous nature of Tiled-C designs avoids this bias as targeted fragments are not enriched more than other fragments within the targeted region. Advantages compared to Hi-C are that Tiled-C can create high-resolution contact matrices of regions of interest at great depth in multiplexed samples for a fraction of the sequencing costs associated with genome-wide high-resolution Hi-C experiments. Other approaches which allow for many-versus-many analysis within regions of interest include methods such as Chromosome Conformation Capture Carbon Copy (5C)^57^, Targeted Chromatin Capture (T2C)^58^, Capture Hi-C (cHi-C)^59^, HYbrid Capture Hi-C (Hi-C^2^)^60^, and Tiled-MCC^61^. An important advantage of Tiled-C compared to these methods is that it allows for high-quality data generation from as few as 2,000 cells^13^.

#### Single-allele multiway analyses

Most 3C methods, including Capture-C, 4C and Hi-C, focus on the analysis of pairwise interactions in cell populations. These methods therefore do not provide information about the higher-order assembly of chromatin structures and their dynamics in individual cells. The long ligation products in 3C libraries contain many ligation junctions between multiple DNA elements in a concatemer. These elements were in close proximity in the cell nucleus at the time of fixation. Therefore, analysis of sequencing reads with multiple junctions allows for the investigation of multi-way chromatin interactions between DNA elements in individual nuclei. Tri-C was developed to identify such multi-way interactions with viewpoints of interest with high sensitivity and at high resolution^11^. By using a restriction enzyme to create small restriction fragments at the viewpoints of interest − usually *NlaIII* − and creating longer sonication fragments, multiple interacting fragments can be analyzed efficiently using high-quality Illumina sequencing. Compared to other recently developed approaches to detect multi-way chromatin interactions, such as chromosomal walks, three-way 4C^62^ and multi-contact 4C (MC-4C)^63,64^, Tri-C offers advantages in throughput, sensitivity, and resolution, as well as careful quantification of interaction frequencies due to robust PCR duplicate filtering^11^. Other recent innovative techniques, such as genome architecture mapping (GAM) and Multiplex-GAM^65,66^, split-pool recognition of interactions by tag extension (SPRITE)^67^, DNA seqFISH+^68^, and single-cell Hi-C^69–74^, also allow for investigation of chromosomal organization in single cells. Since the resolution of these techniques at the moment is limited, these methods have predominantly contributed to our understanding of chromosomal structures in single cells at relatively large-scale, rather than at the level of individual regulatory DNA elements.

### Experimental design

#### Enzyme Selection for Resolution

While theoretically any restriction enzyme could be used in 3C, only a few enzymes efficiently digest chromatin, especially when it is heavily crosslinked. The choice of restriction enzyme for generation of 3C material is the largest determinant of experimental resolution; Capture-C libraries use the 4-base cutters (*Nla*III, *DpnII*) which cut approximately 16-times more frequently than 6-base cutters *(HindIII).* Whilst the higher resolution provided by 4-base cutters allows for distinguishing interactions of nearby elements, deeper levels of sequencing are required to deal with the more complex sequencing libraries. Generally, it is best to perform experiments at high-resolution and select the restriction enzyme based on its cut sites at targets of interest. Since interactions are detected as newly formed ligation junctions between the ends of restriction fragments, the enzyme should be selected based on the proximity of cut sites to the element of interest (< 2 kb linear distance) and the ability to design effective probes for regions of interest.

#### Viewpoint Selection for NuTi Capture-C

Several tools exist for oligonucleotide design for selected viewpoint fragments, including Capsequm2^75^ [http://apps.molbiol.ox.ac.uk/CaptureC/cgi-bin/CapSequm.cgi], HiCapTools^76^ [https://github.com/sahlenlab/HiCapTools], GOPHER^77^ [https://gopher.readthedocs.io] and Oligo^13^ [https://oligo.readthedocs.io/en/latest/index.html]. The targeted fragment should be either overlapping with or very close (<2 kb) to the genomic element of interest and be large enough to accommodate binding of enrichment oligonucleotides (70-120 bp), but not so large that probes are a long way from the element of interest. NuTi Capture-C with a single oligonucleotide per viewpoint is possible; however, this results in lower data depth than with two oligonucleotides − one targeting each end of the fragment. While still providing informative profiles, fragments shorter than 250 bp have been shown to have higher levels of *trans* interactions than longer fragments within the same 3C library^10^, therefore optimal fragment length is 250-1,000 bp. The sequence underlying the oligonucleotides is also an important consideration. Duplication or high sequence similarity (determined using BLAT^78^ and RepeatMasker^79^ with Capsequm2) of oligonucleotide sites will result in off-target pull-down. For some loci (e.g. the alpha and beta globin genes) interaction profiles are still easily interpretable despite duplication, whereas for other genes (e.g. glycophorin encoding genes) high sequence similarity results in data which are harder to interpret; a limitation which is common to most sequencing based 3C methods. Oligonucleotides likely to result in off-target pull down can generally be avoided by selecting an adjacent fragment, or changing restriction enzyme.

#### Oligonucleotide Pool Complexity

**Figure 2:**
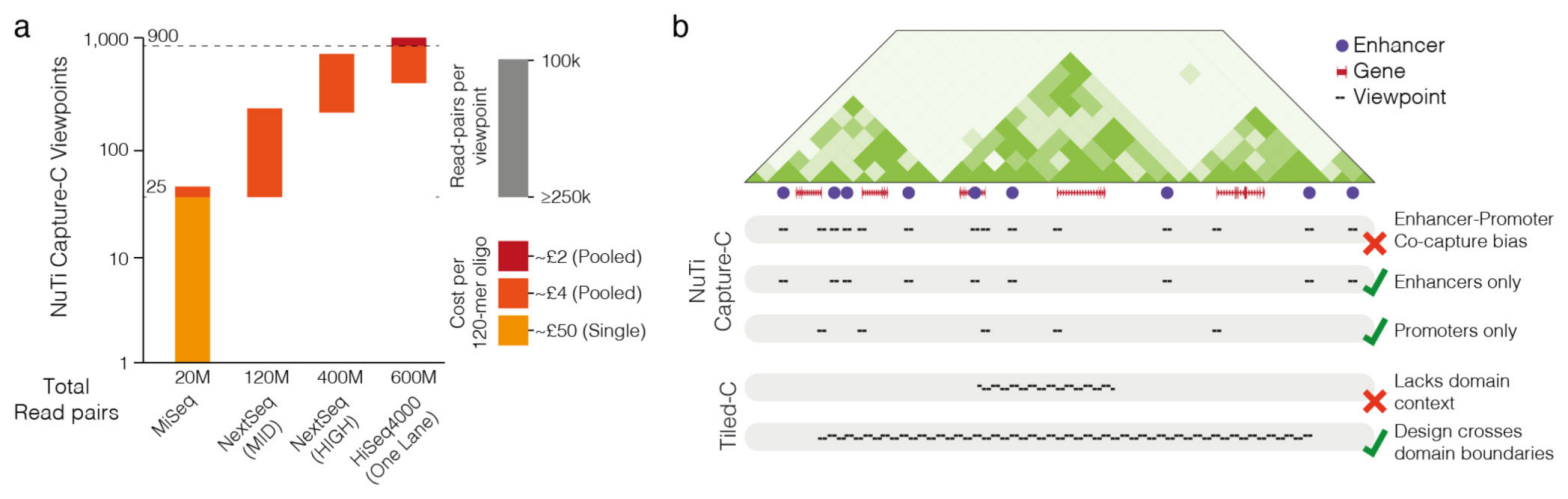
Capture-C design considerations. **a,** Plot of the number of viewpoints that can be sequenced at sufficient depth with the most common Illumina sequencing platforms. Calculations are performed for a NuTi Capture-C experiment with six multiplexed 3C libraries and two oligonucleotides per viewpoint. The range of the bar indicates the number of viewpoints that can be sequenced on the indicated Illumina platforms, with the lower number of viewpoints corresponding to ≥250,000 reads per viewpoint and the higher number of viewpoints to ~100,000 reads per viewpoint. Since capture oligonucleotides can be ordered in pools with a fixed price, a larger number of viewpoints corresponds to substantially reduced costs per oligonucleotide. **b,** When designing pools of oligonucleotides it is important to consider the composition. For many-versus-all approaches, including NuTi Capture-C, pairs of elements that may have interactions of interest (e.g. defining enhancer-promoter interactions) should not be captured simultaneously due to co-capture bias. Instead two pools, targeting only promoters and only enhancers should be used in two separate hybridisation reactions. For contiguous many-versus-many approaches, such as Tiled-C, low-resolution Hi-C can be used to guide selection of the area of interest and ensure domain context (e.g. boundaries and flanking domains) is included.

Several important factors should be considered for combining multiple viewpoints into a single pull-down design. Although it is possible to work with very few oligonucleotides/viewpoints, it can be as cost-efficient to buy pairs of oligonucleotides targeting 25 viewpoints as it is to target only two viewpoints (Fig. 2a). These additional oligonucleotides also increase the total DNA recovered after titrated capture and help to avoid working with very small amounts of DNA. However, increasing the number of viewpoints does increase the total depth of sequencing required. Although it varies depending on library quality and enrichment strategy, a sequencing depth of 100,000-500,000 read pairs per viewpoint per 3C library should be sufficient to identify 20,000 unique reporters for NuTi Capture-C^10^. A single MiSeq run generating 20M paired-end reads should therefore provide sufficient sequencing coverage for 5-25 viewpoints in six 3C libraries. Some analytical tools for calling interactions, such as peaky^80^ and peakC^81^ also benefit from having numerous viewpoints, as this allows for generation of an accurate background model of non-specific polymer interactions.

For targeting of specific disperse elements (NuTi Capture-C, Tri-C), it is important not to simultaneously enrich at two sites whose direct interactions you are interested in, for example co-targeting of a promoter and its cognate enhancers (Fig. 2b), or targeting two promoters which may interact. As all 3C enrichment methods are not 100% efficient, co-targeting is significant source of bias which results in increased observed interaction between targeted sites; see Downes *et al* (2021)^10^ for an experimental and mathematical description of this phenomenon. To avoid this bias, separate enrichments can be performed on aliquots of the same 3C material, targeting, for example, only enhancers and only promoters.

#### Comparative Samples

Capture-C enrichment can be performed on multiplexed samples in a single tube. This approach minimises the technical variation in enrichment, generating highly reproducible profiles for statistical analyses. All of the Capture-C methods are usually performed in triplicate (at least) and can therefore be used to compare different genetic models, or cell types in a single experiment. By performing experiments with triplicates, simple statistical tests (e.g. Student’s t-test) can be used to compare interactions with specific regions, or more advanced approaches (e.g. DESeq2^82^) can be used across entire domains of interaction. 3C interaction profiles from highly related cell-types or throughout differentiation can be remarkably similar. Therefore, it is often beneficial to compare samples with a highly unrelated cell type where elements of interest (e.g. enhancer or promoters) are inactive. It is important to note that there can be considerable technical variability between different cell types in the 3C procedure, which can result in differing levels of background noise (i.e. *trans* interactions) across cell types. Care should be taken to ensure comparative samples have similar noise levels. This can partially be controlled for by normalisation of interaction counts in *cis* rather than to total interactions, as, different levels of *trans* interactions can alter observed proximal interaction frequencies^10^ after normalisation.

#### Tri-C design considerations

Tri-C viewpoints should be located on small (~150-250 bp) restriction fragments generated by the restriction enzyme used for chromatin digestion, which is usually *NlaIII*, since it has a smaller median fragment size compared to *Dpn*II^11^. The ~120 bp capture oligonucleotides should be designed to the middle of the restriction fragments on which the viewpoints of interest are located and repetitive sequences should be avoided.

#### Tiled-C design considerations

Similar considerations as for NuTi Capture-C apply to the design of capture oligonucleotides for Tiled-C. Probes for adjacent restriction fragments in regions of interest can be designed and filtered for repetitive sequences using Oligo^13^ [https://oligo.readthedocs.io/en/latest/index.html]. When determining the extent of regions of interest captured it is useful to use low-resolution Hi-C as a guide for the existence and location of regulatory domains and their boundaries. Both the 3D Genome Browser^83^ [http://3dgenome.fsm.northwestern.edu/view.php] and HiGlass^84^ [https://higlass.io/] provide rich resources of easily accessible Hi-C data in a range of cell-types for this purpose. It is best to be generous in extending the tiled region beyond predicted boundaries for regions of interest to provide an informative regulatory context (Fig. 2b).

### Data analysis

Multiple software packages exist for processing of Capture-C sequencing files. Reads from NuTi Capture-C and LI Capture-C experiments are compatible with HiC-Pro^85^ [https://github.com/nservant/HiC-Pro/releases], capC-MAP^86^ [https://github.com/cbrackley/capC-MAP], CCseqBasic^75^ [https://github.com/Hughes-Genome-Group/CCseqBasicS]. Tri-C data can be analyzed using CCseqBasic or TriC^11^ [https://github.com/oudelaar/TriC] scripts and Tiled-C data can be analyzed using the HiC-Pro pipeline^85^ (with the options for Capture Hi-C analysis) or Tiled-C^13^ https://github.com/oudelaar/TiledC] scripts. Interaction counts can be further processed in a range of 3C-specific tools including, CHiCAGO^87^ [http://functionalgenecontrol.group/chicago], peakC^81^ [https://github.com/deWitLab/peakC], r3Cseq^88^ [http://r3cseq.genereg.net/Site/index.html], FourCSeq^89^ [http://bioconductor.org/packages/release/bioc/html/FourCSeq.html], peaky^80^ [https://github.com/cqgd/pky], CaptureCompare^75^ https://github.com/djdownes/CaptureCompare], and CaptureSee^75^ [https://capturesee.molbiol.ox.ac.uk/]. We find application of peaky, either after processing with CaptureSee^10^ or CHiCAGO^90^ gives highly specific interaction calls.

To facilitate consistent data processing, analysis and interpretation of NuTi Capture-C, Tri-C and Tiled-C data, we developed a computational tool called CapCruncher^91^ [https://github.com/sims-lab/CapCruncher/releases] to analyse all three experiment types. This pipeline utilises python and is both easy to install and run. CapCruncher processes raw fastq files, removes PCR duplicates, identifies reporter reads, and generates a UCSC Genome browser hub with depth normalised tracks for individual replicates and for the mean of replicates. When multiple samples are provided simultaneously, CapCruncher, also generates comparative tracks by subtracting sample means. For Tri-C and Tiled-C, CapCruncher generates visualisation matrices over targeted regions. The CapCruncher pipeline is available on GitHub and Bioconda, and, for testing, a small NuTi Capture-C test dataset can be found on the Gene Expression Ontology database (GSE129378) [https://www.ncbi.nlm.nih.gov/geo/query/acc.cgi?acc=GSE129378]. It should be noted that CapCruncher is under continuous development to add and enhance functionalities. We recommend that users read the corresponding manual [https://capcruncher.readthedocs.io/en/latest/] provided online (the current manual is provided as Supplementary Manual). Below we describe the basic requirements and implementation of CapCruncher.

### Expertise needed to implement the protocol

The experimental processes associated with Capture-C methods are common modern molecular techniques, including: restriction enzyme digestion, proximity ligation, phenol-chloroform DNA extraction, quantitative and standard PCR, gel electrophoresis next-generation sequencing library preparation (including AMPure XP SPRI Bead clean-ups), streptavidin bead pull-down/washes and next-generation sequencing. The equipment for all of these processes (perhaps with exception of a sequencing platform) should be readily available in most research institutes, but where possible, alternatives are suggested. Following sequencing, analysis with CapCruncher, requires basic level unix command line operation which can be easily learnt. However, system administrator rights may be required to install tools, and advanced bioinformatics skills will aid in more complex analyses, such as interaction calling with peaky and other packages.

### Configuration files

Three files are required for successful implementation of the Capture-C methods. For designing oligonucleotide probes, a bed format file (chromosome, start, stop, name) giving single base-pair coordinates to sites of interest is required for Capsequm2. For running of CapCruncher, a bed format file specifying the enriched regions (single fragments for NuTi Capture-C and Tri-C, and extended regions for Tiled-C), and a configuration file specifying the genome, mapping parameters, experimental method, and output directories are required. Examples of all three files are included as Supplementary Data 1-3.

### Limitations

Due to the extremely high efficiency of on-target sequencing afforded by oligonucleotide pull-down, no selection is performed for successful digestion events (unlike Hi-C). Although excluding these relatively inefficient steps reduces the number of cells required for high-resolution data, it does mean that quality control for a high-efficiency digestion is paramount to ensure sequence reads are not wasted. Quality control can be performed either by agarose gel, or more accurately with quantitative real-time PCR (Box 1). Using both methods is optimal. Based on analysis with the latter, 3C libraries should have a minimum 70% digestion for use.

Capture-C methods provide a temporal, population-based snapshot of active chromatin folding processes. To develop a more granular or dynamic perspective of interactions, Capture-C can be complemented with imaging approaches, particularly high-resolution FISH or live-cell imaging^92–97^. The requirement for single-cell suspensions also limits the application of Capture-C methods, since they are not suitable for complex tissues where mixed cell-types cannot be easily separated into pure populations, or for formalin-fixed paraffin embedded (FFPE) samples, such as biopsies and tissue-sections. In these cases, GAM^65,66^ provides a superior ability to separate cell-types of interest and determine interaction dynamics.

3C provides information on chromatin folding, however, it is most informative when it is presented in conjunction with open-chromatin assays (e.g. ATAC-seq^98^, DNase I-seq^99^), and ChIP-seq for epigenetic markers, e.g. promoters (H3K4me3), enhancers (H3K4me1), active-transcription (H3K27ac) and polycomb repression (H3K27me3), and boundary and insulator sites (CTCF).

## Materials

### Biological materials

- *Cells.* Capture-C methods are possible in any eukaryotic species or cell-type where a single-cell
  suspension containing as few as 10,000-20,000 cells can be generated^9,13^. However, if available, using >100,000 will result in data of higher depth and resolution. Successful experiments have been performed previously in fly and chicken, as well as in numerous mouse cell types including embryonic stem cells (ESCs), ter119+ erythroid cells, ESC derived mesoderm, definitive endoderm, neural progenitor cells, mouse embryonic fibroblasts (MEFs [https://scicrunch.org/resolver/RRID:CVCL_4240]), 416B myeloid progenitor cells [https://scicrunch.org/resolver/RRID:CVCL_3983] and J558L B myeloma cells [https://scicrunch.org/resolver/RRID:CVCL_3949]. Capture-C has been widely used in primary human samples, including CD4^+^ T-cells, CD14^+^ Monocytes, HUVEC and CD71^+^ CD235^+^ erythroid cells, as well as human cell lines including lymphoblastoid cell lines (LCL, GM12878 [https://scicrunch.org/resolver/RRID:CVCL_7526]), human ESCs (H1-hESC [https://scicrunch.org/resolver/RRID:CVCL_9771]), lung fibroblasts (IMR-90 [https://scicrunch.org/resolver/RRID:CVCL_0347]) and lung epithelial cells (NCI-H441 [https://scicrunch.org/resolver/RRID:CVCL_1561]), induced pluripotent stem cells (iPSC) and iPSC derived cardiomyocytes, pancreatic beta cells (EndoC-ßH1 [https://scicrunch.org/resolver/RRID:CVCL_L909]), cervical and breast cancer cell lines (MCF-7 [https://scicrunch.org/resolver/RRID:CVCL_0031], MDA-MB-231 [https://scicrunch.org/resolver/RRID:CVCL_0062], HeLa [https://scicrunch.org/resolver/RRID:CVCL_0058]), and leukaemia derived cell lines (K562 [https://scicrunch.org/resolver/RRID:CVCL_0004], SEM [https://scicrunch.org/resolver/RRID:CVCL_0095], RS4;11 [https://scicrunch.org/resolver/RRID:CVCL_0093], THP1 [https://scicrunch.org/resolver/RRID:CVCL_0006]). !CAUTION Cell lines used in your research should be regularly checked to ensure they are authentic and are not infected with mycoplasma.

### Reagents

#### Common Reagents

- PCR Grade Water (Ambion: AM9932)
- Absolute Ethanol (VWR: 20821.330)
- Agencourt AMPure XP SPRI Beads (Beckman Coulter: A63881)
- Qubit dsDNA BR Assay kit (Invitrogen: Q32850)
- D1000 Reagents (Agilent: 50675583)

#### Fixation and 3C library generation

- Formaldehyde, 37% vol/vol (Sigma: 47608-250ML) !CAUTION Formaldehyde is toxic, wear gloves and avoid contact with skin.
- Glycine, 1M (Sigma: G7126)
- PBS (Invitrogen: 10010031)
- Tris pH 8, 1M (Invitrogen: AM9855G)
- NaCl, 5M (Invitrogen: AM9760G)
- Igepal CA-630 (Sigma: I8896)
- cOmplete Protease Inhibitor Cocktail (Sigma: 11873580001)
- SDS, 20% vol/vol (Invitrogen: AM9820)
- Triton-X 100 (Sigma: T8787)
- *DpnII* HC (NEB: R0543M) or *NlaIII* (NEB: R0125L)
- T4 DNA HC Ligase (Life Tech: EL0013)
- Tris-EDTA (TE) Buffer Solution (Sigma: 93302)
- Proteinase K (Thermo Fisher: EO0491)
- RNase (Roche: 1119915)
- Poly ethylene glycol (PEG) 300 (Sigma: 90878-250ML-F)
- Phenol-Chloroform-Isoamylalcohol (PCI) 25:24:1 (Sigma: 77617) !CAUTION Phenol is toxic; avoid skin contact, consider use in a fume hood, dispose of waste appropriately and have PEG 300 easily accessible to treat burns.
- NaOAc pH 5.6, 3M (Invitrogen: AM9740)
- GlycoBlue (Thermo Fisher: AM9515)
- Tris Acetate-EDTA Buffer, TAE (Sigma: T9650)
- Agarose (Sigma: A4718)
- Ethidium Bromide, or equivalent (Invitrogen: 15585011)
- Gel loading dye (NEB: B7024S)
- 1 kb DNA ladder (NEB: N0468S)
- Genomic DNA ScreenTape, *if required* (Agilent: 50675365)
- Genomic DNA Reagents, *if required* (Agilent: 50675366)
- Real time PCR primers (See Table 1, Box 1)
- KAPA Sybr Fast Universal (KAPA: KK4602)

#### Library Indexing

- 3C library (generated in earlier stage)
- NEBNext Ultra II (New England: 7645S/L)
- NEBNext Multiplex Oligos for Illumina Primer set 1 (New England: E7335S/L)
- NEBNext Multiplex Oligos for Illumina Primer set 2 (New England: E7500S/L)
- Herculase II Fusion Polymerase kit (Agilent: 600677)

#### Oligonucleotide pulldown: general reagents

- 1-2 μg of each of six indexed 3C libraries (generated in earlier stage)
- Qubit dsDNA HS Assay Kit (Invitrogen: Q32851)
- High Sensitivity D1000 Reagents (Agilent: 5067 5585)
- High Sensitivity D1000 ScreenTape (Agilent: 5067-5584)
- KAPA Library Quantification Complete Kit, Universal (KAPA: KK4824)
- Mouse COT DNA, *if required* (Invitrogen: 18440016)
- Chicken Hybloc competitor DNA, *if required* (Applied Genetics Laboratories: CHB)
- KAPA Hybrid Enhancer Reagent, *if required* (Roche: 09075763001)
  CRITICAL Cot-1 DNA inhibits nonspecific probe binding. Only Cot-1 DNA specific to the organism of interest is required. For human cells, use either the Cot-1 DNA included in either the HyperCapture kit or the Twist Universal blockers. Hybloc reagents are available for several additional organisms; when no species-specific Cot-1 DNA is available the KAPA Hybrid Enhancer Reagent may be used.

#### Oligonucleotide pulldown: For ssDNA oligonucleotides

- Biotinylated probes (e.g. Sigma HPCL purified oligonucleotides, IDT xGen Lockdown Pool)
- HyperCapture Target Enrichment kit, *includes Human COT DNA* (Roche: 09075810001)
- M-270 Streptavidin Dynabeads (Invitrogen: 65305)
- Oligonucleotide pulldown: For dsDNA oligonucleotides
- Twist NGS Target Enrichment Oligonucleotide Panel (Twist: 100533)
- Twist Hybridization and Wash Kit (Twist: 101025/101026)
- Twist Universal Blockers (Twist: 100767)
- KAPA HiFi HotStart ReadyMix (Roche: KK2601)
- MyOne Streptavidin C1 Dynabeads (ThermoFisher: 65001)

## Equipment

- Thermomixer C (Eppendorf: 2230000049), or equivalent
- Electrophoresis tank and power pack
- Qubit 4 Fluorometer (ThermoFisher: Q33238), or equivalent
- Sonicator, e.g. Covaris M220 or S220 Focused-ultrasonicator, or equivalent
- Quantitative Thermocycler
- Thermocycler
- DynaMag-2 (Invitrogen: 13221D), or equivalent
- 4200 TapeStation (Agilent: G2991AA), or equivalent
- Speedy-Vac vacuum centrifuge, or equivalent but not essential
- Light PhaseLock Gel Tubes (5Prime: 733-2477)
- 96-well optical PCR plate
- Covaris microTUBE AFA Fiber pre-split snap-cap 6x16mm (Covaris: 520045), or equivalent
- D1000 Loading Tips (Agilent: 50675153)
- D1000 ScreenTape (Agilent: 50675582)
- High-quality, non-sticky 1.5 mL Microcentrifuge Tubes (e.g. Sorenson BioScience: 39640T) CRITICAL Use these tubes for hybridisation steps with streptavidin beads (steps 86-109, 138-151).

## Reagent Setup

### Igepal CA-630, 10% vol/vol

Mix 1 mL of Igepal CA-630 with 9 mL of PCR grade water. Store at room temperature (RT: 20-22°C) long-term.

### Triton-X, 20% vol/vol

Mix 2 mL of Triton-X with 8 mL of PCR grade water. Store at RT long-term.

### Fresh lysis buffer

Mix reagents on the day of use. Cool to 4°C on ice or on a roller in a cold room. ?TROUBLESHOOTING

**Table:** 

Reagent	Stock Conc.	Volume	Work Conc.
PCR Grade Water	-	48.4 mL	-
Tris pH8	1 M	500 μL	10 mM
NaCl	4 M	125 μL	10 mM
Igepal CA-630	10% vol/vol	1 mL	0.2% vol/vol
cOmplete Protease Inhibitor Cocktail	-	1 tablet	1×

### Ethanol, 70% vol/vol

Mix 7 mL of absolute ethanol with 3 mL of PCR grade water. Store at RT.

### Ethanol, 80% vol/vol

Prepare fresh on day of use. Mix 8 mL of absolute ethanol with 2 mL of PCR grade water.

## Procedure

CRITICAL The following protocol describes the generation of a single Nuclear 3C library (for use in any Capture-C method) with either *Dpn*II or *Nla*III, followed by indexing, with appropriate information for Tri-C and low-input sample modifications. Prior to oligonucleotide pull-down, uniquely indexed 3C libraries can be pooled for multiplexed capture. The volumes in this section describe a six library experiment (i.e. triplicates for two cell-types/genetic models) but can be scaled as necessary. Oligonucleotide pull-down can be carried out with either ssDNA oligonucleotides (first described for NG Capture-C^5^) or with dsDNA oligonucleotides (first described for Tiled-C^13^) and descriptions for both protocols are provided. A host of tools are available to analyse Capture-C experiments. Instructions are provided for processing of replicate samples with a portable python script, CapCruncher, which can process all three experiment types.

## Viewpoint Preparation

### Oligonucleotide Probe Design

#### Timing 3 h

1. Use Capsequm2, Oligo or an equivalent tool to design appropriate probes for NuTi Capture-C, Tiled-C or Tri-C (see Experimental Design and Fig 1). For Capsequm2 generate a bed file of single base pair regions under the genomic element of interest: tab separated chromosome, start, stop, and viewpoint name (Supplementary Data 1).

2. Load bed file into Capsequm2 [http://apps.molbiol.ox.ac.uk/CaptureC/cgi-bin/CapSequm.cgi], select probe length (70-120 bp) and genome.

3. Proceed with filtering after fragment extraction error check.

4. Use AltSort to select probes passing filtering and download oligonucleotide sequences.

**Table:** 

Parameter	Setting
Duplicates	≤ 2*
Blat Density	≤ 40
G/C Content (%)	≤ 60
Repeats	False

* Often interactions at duplicated genes, e.g. *HBA1*, *HBA2*, can still be understood.

5. Order biotinylated oligonucleotides (either ssDNA or dsDNA) either in individual tubes for custom pooling, or as pre-mixed pools. CRITICAL STEP Unless performing Tiled-C, it is important not to mix two viewpoints that you wish to directly compare interactions for; co-targeting of distal fragments introduces significant bias for interactions between viewpoints compared with adjacent untargeted fragments. For an explanation of this effect, see the Supplementary Note associated with Downes *et al*. 2021^10^. To avoid cross-contamination during production it can be prudent to order on different days or from different suppliers.

CRITICAL STEP LI capture-C, NG Capture-C, NuTi Capture-C, and Tri-C have traditionally been performed with ssDNA oligonucleotides, whereas Tiled-C has been performed with dsDNA oligonucleotides. However, there is no reason why a specific method could not be performed with either ssDNA or dsDNA oligonucleotides, therefore both protocols are described. Follow the appropriate instructions for enrichment using either ssDNA oligonucleotides (step 75-124) or dsDNA oligonucleotides (step 125-162).

### Oligonucleotide Stock Preparation

#### Timing 1 h

6. Reconstitute individual or pools of oligonucleotides following the manufacturer’s instructions or to a stock concentration so that each unique oligonucleotide is stored at ≥1 μM.

7. If oligonucleotides were ordered individually, generate pools of oligonucleotides by mixing in exact 1:1 stoichiometric ratio and store at -20°C until required at step 81 (ssDNA Probes) or step 129 (dsDNA Probes).

## 3C Library Generation

### Formaldehyde fixation

#### Timing 3 h

8. Pre-cool large centrifuge to 4°C. Chill glycine, PBS, and fresh lysis buffer.

9. Collect cells from whole tissue or culture and make single-cell suspensions of 5 x 10^6^ cells in 5 mL of growth media.?TROUBLESHOOTING

10. Add 270 μL 37% vol/vol formaldehyde (2% vol/vol final conc.) and incubate for 10 min at room temperature while tumbling or rotating. ! CAUTION Formaldehyde is toxic; avoid skin contact, consider use in a fume hood and dispose of waste appropriately. CRITICAL STEP Varying levels of formaldehyde fixation can affect digestion efficiency and levels of *trans* ligation^9^. Use bottles within 3 months of opening or single-use ampules.

11. Quench formaldehyde by adding 750 μL 1M cold glycine (125 mM final conc.).

12. Centrifuge for 10 min at 500 xg (4°C), wash pellet by gently re-suspending in 5 mL ice-cold PBS.

13. Centrifuge for 10 min at 500 xg (4°C), gently remove supernatant without disturbing pellet and re-suspend in 5 mL ice-cold lysis buffer.

14. Incubate for 20 min on ice then centrifuge for 15 min at 500 xg (4°C), gently remove supernatant without disturbing pellet and wash by gently re-suspending in 5 mL cold PBS.

15. Centrifuge for 15 min at 500 xg (4°C), gently remove supernatant without disturbing pellet then re-suspend pellet in 215 μL 1× *DpnII* buffer (*DpnII* libraries) or 215 μL 1× CutSmart® buffer (*Nla*III libraries) and transfer to microcentrifuge tube. CRITICAL STEP For low-input samples (≤150,000 cells), to avoid wasting material, resuspend samples in 200 μL buffer and do not generate controls. Digestion efficiency can be directly determined using the 3C library.

PAUSE POINT Either snap freeze and store at -80°C or proceed to digestion.

### Digestion

#### Timing 24 h

16. Set a thermomixer to 37°C and warm nuclei.

17. Set up Digest and Control 1 (Undigested DNA) as per the table below in Safe-Lock microcentrifuge tube.

**Table:** 

Reagent	Digest	Control 1
***Dpn*II digested 3C library**		
Nuclei in 1× *Dpn*II buffer	200 μL	15 μL
PCR-grade water	434 μL	227.5 μL
10× *Dpn*II buffer	60 μL	28.5 μL
20% vol/vol SDS (0.28% final conc.)	10 μL	4 μL
***NlaIII* digested 3C library**		
Nuclei in 1× CutSmart® buffer	200 μL	15 μL
PCR-grade water	393.5 μL	227.5 μL
10× CutSmart® buffer	60 μL	28.5 μL
20% vol/vol SDS (0.28% final conc.)	9.5 μL	4 μL

CRITICAL STEP Add the SDS last to ensure a maximum concentration of 0.28% vol/vol; pre-warming the nuclei avoids SDS precipitation.

18. Shake horizontally at 500 rpm (intermittent shaking: 30s on / 30s off) for 1 h at 37°C on a thermomixer.

19. Add 20% vol/vol Triton X 100 at a final concentration of 1.67% vol/vol. Add 66 μL to *DpnII* digests, 62 μL to *Nla*III digests, and 25 μL to Control 1. CRITICAL STEP Triton-X quenches SDS by forming micelles and is vital to allow restriction enzyme function.

20. Shake horizontally at 500 rpm (intermittent shaking: 30s on / 30s off) for 1 h at 37°C on a thermomixer.

21. Add 10 μL *Dpn*II (500 U) or 25 μL *Nla*III (250 U) to digestion reactions and incubate at 37°C for several hours.

22. Add a further 10 μL *Dpn*II (500 U) or 25 μL *Nla*III (250 U) to digestion reactions and incubate overnight at 37°C.

23. Add a further 10 μL *Dpn*II (500 U) or 25 μL *Nla*III (250 U) and incubate for another 5-6 hours.

### Ligation

#### Timing 24 h

CRITICAL For low-input samples (≤150,000 cells), skip steps 25-27 and do not make a Control 2. Digestion efficiency can be directly determined using the 3C library.

24. Place the digest at 65°C for 20 min to heat inactivate restriction endonuclease. CRITICAL STEP Move directly to a pre-heated 65°C thermomixer and then immediately cool digests on ice to avoid de-crosslinking.

25. Take 100 μL from the digest reaction to make Control 2 (Digested, un-ligated control).

26. Add 200 μL PCR grade water to Control 2 to make up to 300 μL.

27. Store Control 1 and Control 2 at -20 to 4°C until DNA extraction (step 33).

28. On ice, add 500 μL PCR-grade water and 134 μL 10× Ligation buffer to the digest. Mix by pipetting. CRITICAL STEP. For low-input samples (≤150,000 cells), add 400 μL PCR-grade water and 134 μL 10× Ligation buffer.

29. Add 8 μL T4 Ligase (240 U) and incubate on a 16°C thermomixer at 500 rpm (intermittent: 30s on / 30s off) for ~18 hours.

30. Centrifuge ligation reaction at 500 xg for 15 min (RT).

31. Gently remove all of the supernatant without disturbing nuclear pellet. CRITICAL STEP It is important to remove the majority of the ligation buffer as high levels of DTT will interfere with DNA quantification. However, take care not to disrupt the pelleted nuclei.

32. Resuspend the nuclear pellet in 300 μL of TE buffer. CRITICAL STEP. If using column based extraction rather than Phenol-chloroform isoamyl alcohol DNA extraction (see Troubleshooting for Step 37), follow kit instruction rather than resuspending in TE buffer.

### DNA Extraction

#### Timing 18 h

33. Remove Control 1 and Control 2 from storage.

34. Add 5 μL Proteinase K (3 U) to the library and the controls and incubate at 65°C for 4 hours.

35. Cool reactions to 37°C, and add 5 μL RNase (7.5 mU) to the library and the controls. Incubate for 30 min at 37°C on a thermomixer (500 rpm, intermittent: 30s on / 30s off).

36. Prepare three PhaseLock tubes by spinning at 5,000 xg for 2 min (RT).

37. Add 310 μL phenol-chloroform-isoamyl alcohol (PCI) to each tube, close tightly and vortex thoroughly to mix. !CAUTION Phenol is toxic; avoid skin contact, consider use in a fume hood, dispose of waste appropriately and have PEG 300 easily accessible to treat burns. ?TROUBLESHOOTING

38. Transfer DNA/PCI mix to a pre-spun PhaseLock tube and separate by centrifuging for 10 min at 12,600 xg (RT).

39. Transfer the upper layer to a new microcentrifuge tube, avoiding the viscous interface and then add 30 μL of 3M sodium acetate and 1 μL of glycoblue, mix by inversion.

40. Add 900 μL of ice-cold absolute ethanol (75% vol/vol final conc.) and mix thoroughly by inversion. Freeze at -20°C for at least 2 h, overnight precipitation can improve yield. PAUSE POINT DNA precipitation may be stored at -20°C for several days.

41. During the incubation, cool a centrifuge to 4°C and chill 70% vol/vol ethanol on ice.

42. Pellet DNA by centrifuging at 21,000 xg for 30 min at 4°C and discard supernatant. The pellet should be blue in colour due to the dye in the glycoblue.

43. Wash the DNA pellet by adding 1 mL of ice-cold 70% vol/vol ethanol and pellet by centrifuging at 21,000 xg for 30 min at 4°C. Remove the ethanol and repeat ethanol wash for a total of two washes.

44. After the supernatant from the second ethanol wash is discarded use a benchtop centrifuge to collect residual ethanol. Discard this using a pipette.

45. Dry the DNA pellet at room temperature with the lid open (~15-20 min), when the pellet goes clear resuspend by adding 30 μL TE buffer to controls and 140 μL to the 3C library (digestion reaction).

PAUSE POINT The 3C library can be stored at -20°C for several years.

### Quality Control (See Box 1, Fig. 3)

#### Timing 3 h

CRITICAL Unless cell samples are extremely precious or difficult to isolate, only proceed with 3C libraries with >70% digestion efficiency. Unlike Hi-C methods, no enrichment for successful digestion-ligation events is carried out and low digestion efficiency leads to a high proportion of uninformative reads.

46. Make a 1% (wt/vol) agarose gel using 1× TAE and run at a moderate speed (~70 mA) using 1 μL of 1 kb DNA ladder, 15 μL of each control and 5 μL of 3C library. ?TROUBLESHOOTING

47. Use 2 μL of 3C library in a Qubit BR assay to determine DNA concentration. The DNA yield from a normal diploid mouse or human cell is ~6 pg. A standard yield of 60-75% of input DNA is expected, generally >15 μg from 5 x 10^6^ cells.

48. Perform quantitative real-time PCR to determine digestion efficiency using Control 1 and Control 2 with both Cut-site and Fragment primer sets. Using triplicates for each reaction, combine the reagents in a 96-well optical PCR plate as below. A master mix excluding the DNA can be made prior to adding to the plate.

**Table:** 

2× KAPA SYBR	10 μL
ROX	0.4 μL
Primer mix (10 μM each)	0.6 μL
Water	7 μL
DNA (10 ng/μL)	2 μL

?TROUBLESHOOTING

49. Perform quantitative PCR using the following conditions and calculate digestion efficiency.

**Table:** 

**Step 1**	95°C	20 s
**Step 2**	95°C	3 s
**Step 3**	60°C	30 s
**Step 4**	*Go to Step 1* (x40)	

?TROUBLESHOOTING

## Library Indexing

CRITICAL Sequencing adaptors are added by ligation after sonication. Where sonication is not possible, tagmentation can be used for indexing^9,100^, however custom blocking oligonucleotides may be required for capture.

### Sonication

#### Timing 2 h

50. Bring 235 μL of AMPure XP SPRI beads to room temperature in a microcentrifuge tube (set aside).

51. Add 130 μL of 3C library to a Covaris microtube, avoiding making bubbles.

52. Shear DNA to 200 bp with appropriate settings on the available sonicator.

**Table:** 

S220		M220	
**Duty Cycle**	10 %	**Duty Factor**	20 %
**Intensity**	5	**Peak Power**	70
**Cycles per burst**	200	**Cycles per burst**	1,000
**Time**	360 sec	**Average Power**	14.0
**Mode**	Freq. Sweeping	**Time**	130 sec

CRITICAL STEP These settings are a suggested starting point for optimisation. Different sonciators of the same model can have differing resulting size fragments. It is essential to optimise settings with high molecular weight DNA when using a new machine for the first time. This optimisation can be performed with genomic DNA.

CRITICAL STEP To perform Tri-C, sonicate DNA to a mean fragment size of 450 bp.

**Figure 3:**
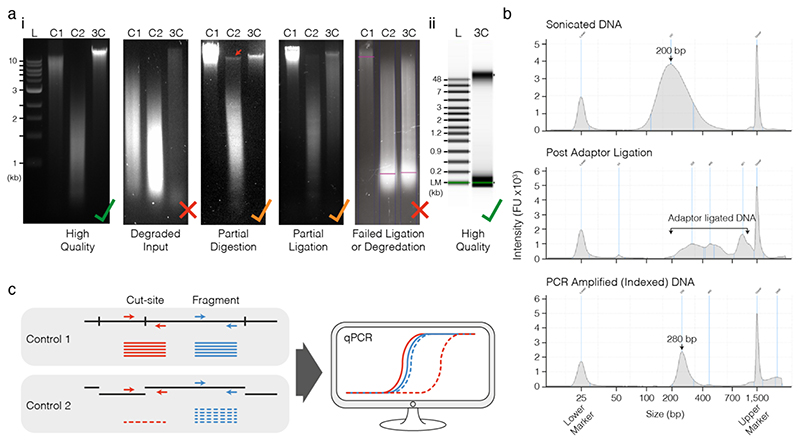
Quality Control of 3C libraries. **a,** Qualitative assessment of undigested input material (Control 1, C1), digested, un-ligated DNA (Control 2, C2) and 3C libraries is performed by electrophoresis in a 1% agarose gel **(i)** or TapesSation Genomic DNA ScreenTape **(ii)**. Examples show a high quality 3C library (green tick) run with a 1 kb DNA ladder (L), moderate quality libraries that can be acceptable for Capture-C (orange ticks), and poor-quality libraries that should not be used (crosses). Note the low proportion of high molecular weight DNA remaining in C2 of the Partial Digestion example (red arrow). Only the 3C library is shown on the TapeStation analysis, note the second band is the Lower Marker (LM) **b,** Tapestation profiles of DNA following sonication, adaptor ligation and PCR amplification provide qualitative assessment of indexing and are used to ensure reactions proceed as expected. **c,** Quantitative assessment of 3C library digestion is performed with real-time PCR using primers that amplify across a restriction digestion site (cut-site, red lines) or within a restriction enzyme fragment (blue lines). Both primer pairs should amplify to the same extent in undigested Control 1 (solid lines), and the difference in amplification in the digested Control 2 (hashed lines) is used to calculate digestion efficiency.

53. Perform an AMPure XP SPRI bead clean-up. Transfer 130 μL of sonicated DNA to 235 μL SPRI beads (1.8×) and mix by pipetting 10 times, incubate at room temperature for 5 min.

54. Place on magnetic stand, discard liquid when clear (~2 min), add 800 μL of fresh 80% vol/vol ethanol without removing from magnetic stand. Incubate for 30 sec and then remove the ethanol. CRITICAL STEP Avoid disturbing beads by running the ethanol down the front of tube.

55. Add a further 800 μL of fresh 80% vol/vol ethanol without removing from magnetic stand. Incubate for 30 sec and then remove the ethanol.

56. Spin down tube on a microcentrifuge and replace on magnetic stand. Remove residual ethanol with a low volume pipette, taking care not to remove any beads.

57. Air dry SPRI beads at room temperature on magnetic stand until matt in appearance. CRITICAL STEP Take care not to over dry the beads as this will result in increased DNA losses; beads will look damp but not glossy when they are ready, overdried beads will develop cracks.

58. Remove from magnetic stand and re-suspend beads in 55 μL of PCR-grade water.

59. Incubate at room temperature for 2 min to elute. Replace on magnetic stand and once clear (~2 min) recover 53 μL.

60. Assess 1 μL of sonicated material using D1000 TapeStation (Fig 3b).

61. Use 2 μL of sonicated 3C library in a Qubit BR assay to determine DNA concentration. ?TROUBLESHOOTING

PAUSE POINT Sonicated DNA can be stored at -20°C for several months.

### End Prep and Adaptor Ligation

#### Timing 3 h

CRITICAL It is important to maintain library complexity by maximising input DNA and minimising DNA losses with the bead clean ups. For this reason, we use a modified protocol for the NEBNext Ultra II kit which only requires a single bead clean-up before indexing. Using 2 μg of human DNA is equivalent to ~5× 10^5^ cells, which can each provide four interactions per viewpoint (two per fragment per allele). The same amount of Drosophila DNA is equivalent to ~7× 10^6^ cells. When ≤1 μg is available for indexing, End Prep and Adaptor Ligation can be performed as described in the product manual.

CRITICAL This protocol is a modified version of the NEB Ultra II indexing protocol. Be aware of changes to composition of kit reagents and workflow.

62. In a PCR tube, dilute up to 2 μg of DNA to 50 μL in PCR-grade water and add 7 μL 10× End Prep Buffer, and 3 μL End Prep Enzyme then mix by pipetting.

63. Incubate End Prep reaction in a thermocycler for 45 min at 20°C, followed by 30 min at 65°C (lid set to 75°C).

64. Add 30 μL Ultra II Ligation Master Mix, 7 μL NEBNext Adaptor and 1 μL Ligation Enhancer, then mix by pipetting and incubate in a thermocycler for 30 min at 20°C (lid off).

65. Add 3 μL of USER™ Enzyme, mix by pipetting and incubate in a thermocycler for 30 min at 37°C (lid 47°C).

66. During the final incubation, bring 180 μL of AMPure XP SPRI beads to room temperature.

67. Perform an SPRI bead clean-up as described at steps 53-59 with 180 μL of AMPure XP SPRI beads. Elute in 59 μL of PCR-grade water and recover 28.5 μL into two PCR tubes.

### PCR Addition of Indices

#### Timing 2 h

68. Bring 180 μL of AMPure XP SPRI beads to room temperature (set aside until step 71).

69. To each PCR tube with 28.5 μL of adaptor ligated DNA add indexing reagents with index specific primers to allow pooling with other samples of interest.

**Table:** 

Adaptor Ligated library	28.5 μL
NEB Universal primer	5 μL
NEB Index primer	5 μL
Herculase II 5× buffer	10 μL
dNTP	0.5 μL
Herculase II polymerase	1 μL

70. Mix by pipetting and amplify DNA using the settings below for a total of six cycles of amplification.

**Table:** 

**Step 1**	98°C	30 s
**Step 2**	98°C	10 s
**Step 3**	65°C	30 s
**Step 4**	72°C	30 s
**Step 5**	*Go to Step 2*	
**Step 6**	72°C	5 min
**Step 7**	4°C	Hold

71. Combine PCR reactions and perform an AMPure XP SPRI bead clean-up as described at steps 53-59 using 180 μL of AMPure XP SPRI beads. Elute in 55 μL of PCR-grade water and recover 53 μL into a new microcentrifuge tube.

72. Assess 1 μL of indexed material using D1000 TapeStation to ensure increase in fragment size (Fig. 3b).

73. Quantify 2 μL of indexed library using Qubit dsDNA BR assay kit.

PAUSE POINT Indexed 3C DNA can be stored at -20°C for several years.

## Capture Enrichment

74. Perform oligonucleotide pull down of target fragments using either single stranded oligonucleotides (ssDNA Probes, steps 75 - 124) or double stranded oligonucleotides (dsDNA Probes, steps 125 - 162).

### Hybridisation (ssDNA Probes)

#### Timing 4 d

CRITICAL Capture-C methods are highly adaptable for multiplexing any number of libraries of interest, and triplicates of each sample (e.g. cell-type, genetic model, treatment, timepoint) are highly recommended. The instructions here are for a standard three-versus-three experiment which permits statistical comparison of interactions. For HyperCapture reagents (ssDNA probes) the maximum number of libraries per tube is 6. For larger designs, pool all libraries then split equivalent amounts of DNA across multiple tubes and scale reaction volumes accordingly.

CRITICAL This protocol is a modified version of the Roche HyperCapture streptavidin pull-down protocol. Be aware of changes to composition of kit reagents and workflow.

75. Heat vacuum centrifuge to 40-50°C.

76. In a PCR tube, combine 1-2 μg from each of six uniquely indexed samples (from step 71) 1:1 by mass, then add 30 μg of species-specific Cot-1 DNA (30 μL of 1 mg/mL stock, 5 μL per library). CRITICAL STEP Cot-1 DNA blocks repetitive elements and is species specific, when a species-specific product is not available, KAPA Hybrid Enhancer Reagent (Roche) can be used.

77. Desiccate in a vacuum centrifuge with tube lids open until sample is completely dry. ?TROUBLESHOOTING

78. To the DNA add 40.2 μL of Universal Enhancing Oligonucleotides (6.7 μL per library) and mix by pipetting. CRITICAL STEP DNA is at a very high concentration and may be sticky so take care to eject all liquid from the pipette tip.

79. Add 84 μL Hybridization buffer (14 μL per library) and 36 μL of Hybridization Component H (6 μL per library), mix carefully by pipetting, briefly centrifuge then incubate at room temperature for 2 min.

80. Replace all buffers and blocking reagents in the freezer to avoid contamination with hybridization oligonucleotides.

81. Defrost oligonucleotide stocks (from step 7), make at least 32 μL of working concentration oligonucleotides by diluting pools to at an optimum titrated concentration (see Box 2).

82. Add 27 μL of diluted oligonucleotides to hybridisation mixture (4.5 μL per library) and mix carefully by pipetting. Briefly centrifuge to collect at bottom of tube. Store remaining oligonucleotides at - 20°C until used in double capture (step 118).

83. Program a thermocycler, to incubate at 95°C for 5 min then hold at 47°C indefinitely (lid 105°C). Add hybridization mixture.

84. Label PCR machine to prevent it being inadvertently turned off and incubate capture reaction at 47°C for 18-22 h.

### Streptavidin Bead Binding (ssDNA Probes)

#### Timing 2 h

85. Heat a thermomixer to 47°C.

86. Bring 300 μL M-270 streptavidin dynabeads to room temperature (50 μL per library) in a low affinity tube. ?TROUBLESHOOTING

87. Prepare Wash buffers, unless stated leave at RT until required:

**Table:** 

Buffer	Buffer volume	Water volume
2.5× Bead Wash buffer	600 μL	900 μL
10× Stringent Wash buffer	120 μL	1,080 μL
10× Wash buffer I	93 μL	837 μL
10× Wash buffer II	60 μL	540 μL_798_
10× Wash buffer III	60 μL	540 μL_799_

CRITICAL STEP If any precipitate is seen in concentrated wash buffers heat to 37°C and ensure complete resuspension prior to making 1 × mixtures.

88. Place 1× Stringent Wash buffer at 47°C.

89. Aliquot 330 μL of 1 × Wash buffer I (55 μL per library) in a fresh tube and place at 47°C.

90. Place beads on a magnetic stand; remove liquid once clear (30 s).

91. Add 600 μL of 1 × Bead Wash buffer (100 μL per library) and vortex to re-suspend the beads, spin briefly. Replace on magnetic stand; remove liquid once clear (30 s).

92. Repeat step 91 for a total of two washes.

93. Remove tube from the magnetic stand and re-suspend the beads in 300 μL of 1 × Bead Wash buffer (50 μL per library).

94. Replace beads on magnetic stand.

95. Working quickly, remove Bead Wash buffer from streptavidin beads and transfer the entire ~185 μL hybridisation reaction from Step 84 (31.2 μL per library) to the streptavidin beads and mix by pipetting.

96. Place on the 47°C thermomixer (600 rpm) and incubate for 45 min to allow probes to bind to the beads. CRITICAL STEP The beads may settle out during hybridisation, resuspend by pipetting after 5 min but take care not to lose too many beads in the tip due to their affinity for plastic.

97. Add 300 μL of heated 1 × Wash buffer I (50 μL per library) to the bead-bound DNA and mix by vortexing for 10 s.

98. Perform a quick spin, then place in magnetic stand and discard all the liquid when clear. Remove from magnetic stand, add 600 μL of heated 1 × Stringent Wash buffer (100 μL per library) and mix by vortexing.

99. Incubate on a thermoxmixer for 5 mins at 47°C (600 rpm), then briefly centrifuge to remove any liquid from lid.

100. Place in magnetic stand and discard all the liquid when clear (30 s). Remove from magnetic stand, and perform a second stringent wash with 600 μL of heated 1 × Stringent Wash buffer (100 μL per library).

101. Incubate on a thermoxmixer for 5 mins at 47°C (600 rpm), then briefly centrifuge to remove any liquid from lid.

102. Place in magnetic stand and discard all the liquid when clear (30 s). Remove from magnet and add 600 μL of room temperature 1× Wash Buffer I (100 μL per library).

103. Mix by vortexing for 10 s, briefly spin in benchtop microcentrifuge to remove any liquid from lid, then incubate at room temperature for 1 min.

104. Place in magnetic stand and discard all the liquid when clear (30 s). Remove from magnet and add 600 μL of room temperature 1× Wash Buffer II (100 μL per library).

105. Mix by vortexing for 10 s, briefly spin in benchtop microcentrifuge to remove any liquid from lid, then incubate at room temperature for 1 min.

106. Place in magnetic stand and discard all the liquid when clear (30 s). Remove from magnet and add 600 μL of room temperature 1× Wash Buffer III (100 μL per library).

107. Mix by vortexing for 10 s, briefly spin in benchtop microcentrifuge to remove any liquid from lid, then incubate at room temperature for 1 min.

108. Place in magnetic stand and discard all the liquid when clear (30 s).

109. Remove from the magnetic stand and resuspend beads in 240 μL PCR grade water (40 μL per library).

PAUSE POINT DNA is not eluted but amplified off the beads, either store the bead bound DNA at -20°C or proceed to amplification.

### PCR Amplification (ssDNA Probes)

#### Timing 2 h

110. Bring 540 μL of AMPure XP beads to room temperature (90 μL per library) and set aside for step 114.

111. To 120 μL of bead bound DNA in water add 150 μL of 2× KAPA HiFi HotStart ReadyMix (25 μL per library) and 30 μL of Post-Capture PCR Oligos (5 μL per library) and mix by pipetting.

112. Aliquot 50 μL of PCR mix into each of six PCR tubes and perform PCR using the following settings with a total of 10-14 cycles of amplification.

**Table:** 

**Step 1**	98°C	45 s
**Step 2**	98°C	15 s
**Step 3**	60°C	30 s
**Step 4**	72°C	30 s
**Step 5**	*Go to Step 2*	
**Step 6**	72°C	60 s
**Step 7**	4°C	Hold

113. Pool six reactions in a microcentrifuge tube and place on a magnetic stand.

114. When clear (30 s), transfer supernatant to a new microcentrifuge tube containing 540 μL of AMPure XP beads (90 μL per library) and perform bead clean up as per steps 53-59 using 540 μL of AMPure XP SPRI beads. Elute into 56 μL of PCR-grade water, recovering 53 μL.

115. OPTIONAL Confirm size of amplified DNA using a high sensitivity D1000 tapestation.

116. Use 2μL of amplified material in a Qubit dsDNA HS assay kit to quantify the DNA. ?TROUBLESHOOTING

117. Repeat amplification (steps 112−114) on the remaining volume of DNA bound streptavidin beads and combine DNA from both amplifications.

### Double Capture (ssDNA Probes)

#### Timing 2 d

CRITICAL When using optimally titrated probes, double capture increases the on-target sequencing efficiency by 2-3 fold over single capture. The amount of DNA recovered after single capture is generally <2 μg so capture is performed as described for a single library using all of the recovered material. For Tiled-C, the high density of probes leads to an extremely high efficiency enrichment and a second capture is not required. If performing Tiled-C proceed to *Sequencing and Analysis* (step 163). Some users have also found that single round of capture at 55°C rather than 47°C can provide high specificity, however this has not been robustly tested.

118. Use 2 μL of amplified material from Step 117 in a Qubit dsDNA HS assay kit to quantify the DNA.

119. Perform *Hybridisation* (*steps 75-84*) as described using volumes for a single library.

120. Perform *Streptavidin Bead Binding* (*steps 85-109*) as described using volumes for a single library and preparing buffers as below. Unless stated otherwise leave at RT until required.

**Table:** 

Buffer	Buffer volume	Water volume
2.5× Bead Wash buffer	100 μL	150 μL
10× Stringent Wash buffer	20 μL	180 μL
10× Wash buffer I	16 μL	144 μL
10× Wash buffer II	10 μL	90 μL
10× Wash buffer III	10 μL	90 μL

121. Place 1 × Stringent Wash buffer at 47°C.

122. Aliquot 60 μL of 1 × Wash buffer I in a fresh tube and place at 47°C.

123. Following the washes resuspend the Streptavidin beads in 20 μL PCR grade water.

124. Perform a single *PCR Amplification* (*steps 110-117*) as described using volumes for a single library with the following adjustments. Elute DNA off beads in 26 μL of PCR-grade water and recover 23 μL. Post amplification size evaluation is not optional and should be performed with standard sensitivity reagents. We recommend using a D1000 Tapestation. Perform DNA quantification with standard sensitivity reagents. We recommend using 2 μL of library in the Qubit dsDNA BR assay kit.

PAUSE POINT Captured DNA may be stored at -20°C for several months until sequencing (step 163).

### Hybridisation (dsDNA Probes)

#### Timing 1.5 d

CRITICAL Capture-C methods are highly adaptable for multiplexing any number of libraries of interest, and triplicates of each sample (e.g. cell-type, genetic model, treatment, timepoint) are highly recommended. The instructions here are for a standard three-versus-three experiment which permits statistical comparison of interactions. For Twist reagents (dsDNA probes) the maximum number of libraries per tube is 8. For larger designs, pool all libraries then split equivalent amounts of DNA across multiple tubes and scale reaction volumes accordingly.

CRITICAL This protocol is a modified version of the Twist target enrichment protocol. Be aware of changes to composition of kit reagents and workflow.

125. Heat a vacuum centrifuge to 40-50°C.

126. In a PCR tube, combine 375-500 μg of six uniquely indexed 3C libraries (from step 71) 1:1 by mass (1,500 μg from up to four libraries per reaction; two reactions can be performed in a single tube or split over two tubes). CRITICAL STEP Use high quality PCR tubes to avoid loss through evaporation during hybridisation.

127. Desiccate in a vacuum centrifuge at 40-50°C with tube lids open until sample is completely dry.

PAUSE POINT Dried DNA may be stored at -20°C.

128. Thaw Hybridization Mix, Hybridization Enhancer, Blocker Solution and Universal Blockers on ice. Once reagents are thawed, vortex briefly to mix components and spin in a microcentrifuge. If precipitate is observed, heat buffers until it is dissolved.

**Figure 4:**
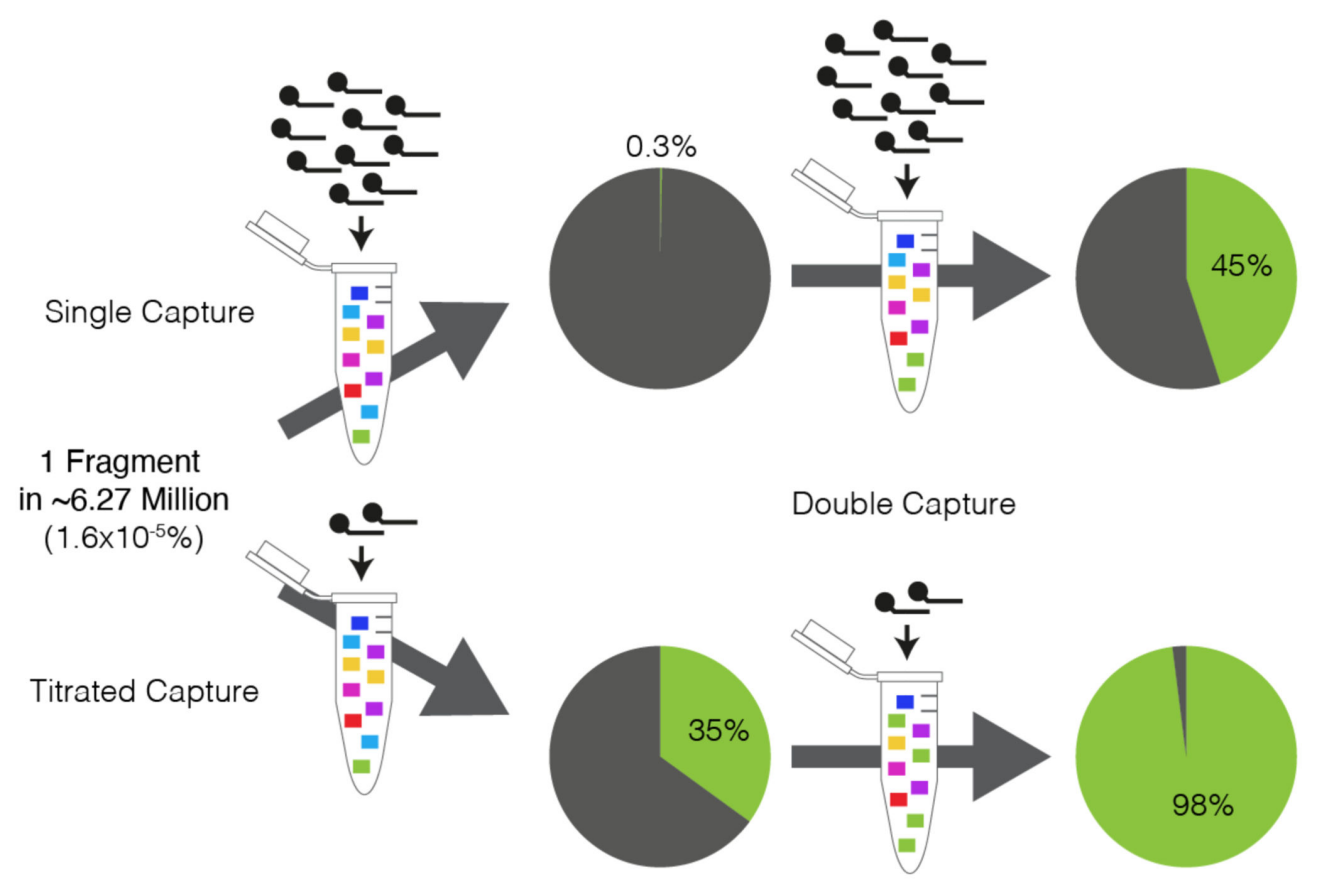
Adaptations for High-specificity Sequencing. Systematic optimization has determined the effect of repeated rounds of oligonucleotide pull-down (Single and Double capture) as well as probe concentration (Titrated capture) on the percentage of reads containing target fragments in mammalian genomes (shown in green).

129. Defrost oligonucleotide stocks (from step 7), make at least 15 μL of working concentration oligonucleotides by diluting pools to at an optimum titrated concentration (see Box 2, Fig. 4 and Table 2). If oligonucleotides are ordered from Twist, follow manufacturer’s recommendations. Store excess probes at -20°C for double capture.

130. In a PCR tube, combine 40 μL Hybridisation mix (20 μL per reaction), 8 μL of capture oligonucleotide and 8 μL of PCR-grade water (4 μL of each per reaction) and mix by pipetting. CRITICAL STEP The hybridisation buffer is very viscous so pipette slowly to ensure accuracy.

131. Resuspend the dried indexed 3C libraries by adding 10 μL Blocker Solution, or species-specific Cot-1 DNA, (5 μL per reaction) and 14 μL Universal Blocker (7 μL per reaction), then carefully mix with a pipette.

132. Denature dsDNA capture Probe Mix by heating to 95°C for 2 min in a thermocycler (lid 105°C), then immediately cool on ice for 5 min.

133. While the Probe Mix is cooling on ice, heat the tube containing the resuspended indexed 3C library pool at 95°C for 5 minutes in a thermal cycler with the lid at 105°C.

134. Equilibrate both the probe solution and resuspended indexed 3C library pool to room temperature on the benchtop for 3 minutes.

135. Carefully mix the room temperature Probe Mix, transfer all 56 μL (28 μL per reaction) to the room temperature indexed 3C Library / Blocker Mix, and mix carefully by pipetting.

136. Add 60 μL of Hybridisation Enhancer (30 μL per reaction). Briefly centrifuge to ensure all solution is collected at the bottom of the PCR tube.

137. Incubate hybridisation reaction at 70°C for 16 h with lid at 85°C.

### Streptavidin Bead Binding (dsDNA Probes)

#### Timing 2 h

138. Heat a thermomixer to 48°C. Bring 200 μL MyOne Streptavidin C1 Dynabeads to room temperature (100 μL per reaction) in a low affinity tube. ?TROUBLESHOOTING

139. Bring 1.6 mL Binding buffer (800 μL per reaction), 400 μL Wash buffer 1 (200 μL per reaction) to room temperature and heat 1.4 mL Wash buffer 2 to 48°C (700 μL per reaction). CRITICAL STEP If any precipitate is seen in Binding buffer, Wash buffer 1 or Wash buffer 2, heat to 48°C until dissolved.

140. Add 400 μL of Binding buffer (200 μL per reaction) to streptavidin beads and mix thoroughly by pipetting, place in a magnetic stand until clear (1 min), and discard the supernatant without disturbing beads.

141. Repeat Binding buffer wash of streptavidin beads (step 140) two times for a total of three washes.

142. After the third and final wash, remove from stand and resuspend in 400 μL of Binding buffer (200 μL per reaction).

143. Remove hybridisation reaction from 70°C thermocycler and quickly transfer all 140 μL (70 μL per reaction) to streptavidin beads in Binding buffer. Incubate at room temperature for 30 min with gentle mixing (on a mixer, shaker, rocker, or rotator) to ensure solution stays homogenised.

144. Briefly centrifuge the Binding reaction to collect the material at the bottom of the tube and place on magnetic stand. When solution is clear (1 min) discard the entire supernatant without disturbing the pellet.

145. Remove from rack, add 400 μL of Wash buffer 1 (200 μL per reaction) and mix by pipetting.

146. Briefly centrifuge to collect the material at the bottom of the tube and transfer the entire volume to a new tube. Place on magnetic stand and when the solution is clear (1 min) discard the entire supernatant without disturbing the pellet.

147. Remove from rack, add 400 μL of 48°C Wash buffer 2 (200 μL per reaction), mix by pipetting and incubate at 48°C for 5 min.

148. Briefly centrifuge to collect the material to the bottom of tube and place on magnetic stand. When solution is clear (1 min) discard the entire supernatant without disturbing the pellet.

149. Repeat heated Wash buffer 2 wash (steps 147-148) two times for a total of three washes.

150. After the third and final wash, collect residual buffer with a low volume pipette. Proceed immediately to the next step and do not allow the beads to dry.

151. Remove from the magnetic stand and resuspend in 90 μL of PCR-grade water (45 μL per reaction). Store on ice in preparation for PCR amplification.

### PCR Amplification (dsDNA Probes)

#### Timing 2 h

152. Bring 360 μL of AMPure XP beads to room temperature (180 μL per reaction), set aside for step 157.

153. Thaw KAPA HiFi HotStart ReadyMix and Amplification Primers on ice and mix.

154. To the streptavidin bead bound DNA add 100 μL of KAPA HiFi HotStart ReadyMix (50 μL per hybridisation reaction) and 10 μL of Amplification Primers (5 μL hybridisation reaction) and mix by pipetting.

155. Aliquot 50 μL of PCR mix into each of four PCR tubes (two per hybridisation reaction) and perform PCR with a total of 10-14 cycles of amplification.

**Table:** 

**Step 1**	98°C	45 s
**Step 2**	98°C	15 s
**Step 3**	60°C	30 s
**Step 4**	72°C	30 s
**Step 5**	*Go to Step 2*	
**Step 6**	72°C	60 s
**Step 7**	4°C	Hold

156. Pool four reactions in a microcentrifuge tube and place on a magnetic stand.

157. When clear (30 s), transfer supernatant to a new microcentrifuge tube containing 360 μL of AMPure XP beads (180 μL per hybridisation reaction) and perform bead clean-up as per step 53-59 using 360 μL of AMPure XP SPRI beads. Elute into 56 μL of PCR-grade water and recover 53 μL.

158. OPTIONAL Confirm size of amplified DNA using a high sensitivity D1000 tapestation.

159. Use 2 μL of amplified material in a Qubit dsDNA HS assay kit to quantify the DNA. ?TROUBLESHOOTING

### Double Capture (dsDNA Probes)

#### Timing 2 d

CRITICAL When using optimally titrated probes, double capture increases the on-target sequencing efficiency by 2-3 fold over single capture. The amount of DNA recovered after single capture is generally <2 μg so capture is performed as described for a single library using all of the recovered material. For Tiled-C, the high density of probes leads to an extremely high efficiency enrichment and a second capture is not required. If performing Tiled-C proceed to *Sequencing and Analysis* (step 163).

160. Perform *Hybridisation* (*steps 125-137*) as described using volumes for a single reaction.

161. Perform *Streptavidin Bead Binding* (*steps 138-151*) as described using volumes for a single reaction.

162. Perform *PCR Amplification* (*steps 152-159*) as described using volumes for a single reaction.

PAUSE POINT Captured DNA may be stored at -20°C for several months until sequencing (step 163)

## Sequencing and Analysis

### Sequencing

#### Timing 2 d

163. Using the measured DNA concentration, make a 10 nM dilution of amplified captured DNA from Step 124 or 162.

164. Perform accurate library quantification of the 10 nM dilution using quantitative PCR with size correction. We recommend using KAPA Library Quantification Kit with 1:10,000 and 1:20,000 dilutions.

165. Dilute DNA to appropriate concentration for sequencing (generally 4 nM) and sequence with paired-end reads. Libraries should be sequenced to a depth of 1-5×10^5^ reads per viewpoint per sample for NuTi Capture-C, 1-10×10^6^ reads per viewpoint per sample for Tri-C, and 3-5×10^6^ reads per Mb per sample for Tiled-C, which is sufficient for 5 kb resolution. CRITICAL STEP Using long reads (150 bp) allows the reconstruction of sequencing fragments. From these fragments it is possible to detect restriction digestion sites and *in silico* digest the chimeric reads generated by 3C. This step is essential for Tri-C experiments where multi-way interactions are detected, but not for Tiled-C and NuTi Capture-C where using shorter reads (40-75 bp) can reduce sequencing costs.

### CapCruncher analysis

#### Timing ~1 d. Will vary depending on viewpoint number and sequencing depth

CRITICAL In this section, we provide a step-by-step description of how to use the CapCruncher^91^ pipeline using triplicate many-verus-all capture of the *HBA1*, *HBA2*, *HBB*, *HBD*, *MYC* and *SLC25A37* genes in human erythroid and ES cells^10^ (GSE129378). Installation (step 166) needs only be implemented once. In this walk-through we assume that a Conda environment on a Linux operating system is in use. Full descriptions for using the software can be found on the GitHub page (https://github.com/sims-lab/CapCruncher/). Modifications may be required in the commands below when using different operating systems. Key difference for analysing Tiled-C and Tri-C data are highlighted, please refer to the software manuals and relevant GitHub pages for full documentation. Commands starting with ‘>’ are executed in the command line.

166. Install CapCruncher using Bioconda.


> conda create -n cc capcruncher


167. If appropriate, prepare sequence fastq files by concatenating multiple lanes and then compress using gzip.


> zcat hESC_repl_L00l_Rl.fastq.gz hESC_L002_Rl.fastq.gz | gzip > hESC_rep1_Rl.fastq.gz


168. Make a directory where the analysis will be carried out.


> mkdir captureC_experimentl



> cd captureC_experimentl


169. Copy or generate symbolic links for all samples for analysis.


> cp /path/t0/fastq/hESC_rep1_R1.fastq.gz


OR


> In -s /full/path/to/fastq/file/hESC_repl_Rl.fastq.gz


170. Prepare a tab separated 4-column bed file of viewpoints (viewpoints.hg38.bed, Supplementary Data 2) with chromosome, fragment start, fragment stop and viewpoint name. When analysing Tiled-C data, provide the start and end co-ordinates for the targeted region.

**Table:** 

> nano viewpoints.hg38.bed			
chr16	226254	227156	HBA1
chrl6	222450	223352	HBA2
chr8	l28748253	l28748439	MYC
chr8	23385780	23386686	SLC25A37
chr11	5247977	5248607	HBB
chr11	5255391	5256556	HBD

171. Prepare a config file (config.yml, Supplementary Data 3) specifying mapping genome, restriction enzyme, path to viewpoints file, public file folder. Analysis method should be specified as “capture”. When analysing Tiled-C data use “tiled” and for Tri-C data use “tri”.


> wget https://raw.githubusercontent.com/sims-lab/CapCruncher/master/config.yml



> nano config.yml


172. Run the pipeline.?TROUBLESHOOTING


> conda activate cc



> capcruncher pipeline make


173. Combine the http server URL with the public path specified in config.yml (e.g. (http://userweb.molbiol.ox.ac.uk/datashare/project/fgenomics/publications/Downes_2021_NuTi_Protocol/Downes_2021_NuTi_Protocol.hub.txt) and load this into the UCSC Genome Browser track hub “My hubs” tab.

174. In the My hubs tab click on the hub description to visualise analysis statistics or go to Genome Browser to view data.

### Timing

#### Viewpoint Preparation

Step 1-5, Oligonucleotide Probe Design: 3 h

Step 6-7, Oligonucleotide Stock Preparation: 1 h

### 3C library generation

#### Day 1-5

Step 8-15, Formaldehyde Fixation: 3 h

Step 16-23, Digestion: 24 h

Step 24-32, Ligation: 24 h

Step 33-45, DNA Extraction: 24 h

Step 46-49, Quality Control: 3 h

### Library Indexing

#### Day 6

Step 50-61, Sonication: 2 h

Step 62-67, End Prep and Adaptor Ligation: 3 h

Step 68-73, PCR Addition of Indices: 2 h

### Capture Enrichment − ssDNA Probes

#### Day 7-12

Step 75-84, Hybridisation: 4 d

Step 85-109, Streptavidin Bead Binding: 2 h

Step 110-117, PCR Amplification: 2 h

Step 118-124, Double Capture: 2 d

### Capture Enrichment − dsDNA Probes

#### Day 7-12

Step 125-137, Hybridisation: 1.5 d

Step 138-151, Streptavidin Bead Binding: 2 h

Step 152-159, PCR Amplification: 2 h

Step 160-162, Double Capture: 2 d

### Sequencing and Analysis

#### Day 13-16

Step 163-165, Sequencing: 2 d

Step 166-174, CapCruncher processing: 2-48 h

### Anticipated Results

**Figure 5:**
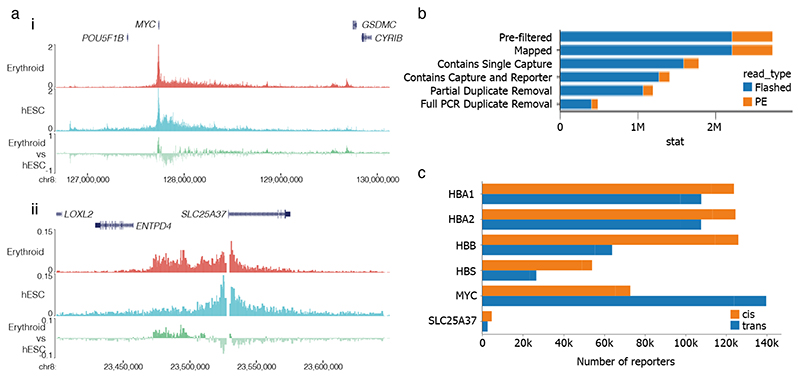
Anticipated results. **a,** NuTi Capture-C profiles exported from the UCSC Browser hub for *MYC* (i) and *SLC25A37* (ii) in Erythroid cells and H1 Human embryonic stem cells (hESCs), with a comparison subtraction track^10^. The *MYC* promoter shows tissue-specific interactions over a 3 Mb scale (chr8:126,675,000-130,130,000, hg38). The *SLC25A37* profiles were generated from replicates of three high-quality 3C libraries with only 3,000 *cis*-reporters each. They still show an easily interpretable 3C interaction plot with erythroid specific interactions (chr8:23,400,000-23,650,000, hg38). **b,** Mapping and filtering statistics with counts of read pairs following FLAShing and *in silico* digestion. Reads that didn’t FLASh are treated as paired end (PE). **c,** Counts of unique reporters for capture from a hESC separated into *cis* and *trans* mapping reads.

A successful NuTi Capture-C profile will provide a near continuous distribution of reported interactions around the central viewpoint, with >20,000 unique *cis*-interaction events (Fig. 5a). When comparing cell-types or genetic models, a CapCruncher run will also generate comparison tracks for identification of tissue-specific interactions. These interaction profiles can be further processed with a range of tools to identify statistically significant interactions.

#### Quality control

The CapCruncher output provides comprehensive quality control metrics as an html webpage to allow users to judge the success, or shortcomings, of a given experiment or 3C library. This report includes fastq PCR duplicate content, adapter trimming, fast length alignment of short reads (FLASh) percentage, *in silico* digestion statistics, alignment statistics, and capture statistics (Fig. 5). Generally Capture-C libraries are deeply sequenced to ensure maximum detection of all possible ligation events. Deep sequencing results in a high number of duplicate reads, generally 25-50%, though if very deeply sequenced up to 90% may be observed. Sequencing is preferably, though not essentially, carried out with long reads to facilitate FLAShing for identification of restriction enzyme cut sites, with 150-bp paired-end reads generating ~90% flashed reads, of which ~70% will contain *DpnII* sites, though this will vary with different sonication conditions and oligonucleotide probe length^10^.

The key metrics of a Capture-C experiment are the alignment filtering statistics, where capture efficiency and reporter content are measured (Fig. 5b). Titration of capture oligonucleotides will result in 80-98% of mapped reads containing a target capture fragment. Lower percentages may indicate that probes were not used at the correct concentration, hybridisation conditions/buffers were not optimal, or that off target capture was a significant factor. Of the capture containing fragments, 60-80% should also contain a reporter. A portion of the reads filtered out at this step are contained in the capture-adjacent fragment, arising from religation of DNA into its original confirmation. Unlike Hi-C, the Capture-C methods do not perform enrichment

for successful digestion and ligation events. Therefore, unflashed capture-containing fragments may lack a restriction enzyme site, which occurs when a sonicated fragment is entirely contained within the viewpoint restriction fragment, or contains a ligation junction with its adjacent restriction fragment. Poor digestion efficiency of a 3C library will significantly increase the proportion of these fragments, lowering the informative proportion of reads. Reporter statistics provide the per viewpoint count of reporters in both *cis* and *trans* (Fig. 5c). High quality 3C libraries and capture provide over 100,000 *cis* reporters per viewpoint, which should make up >60% of all reporters, however, the *cis/trans* ratio is variable amongst viewpoints and can be affected by nuclear positions and fragment length. It’s important to note that outlier viewpoints that have many more *trans* interactions than other veiwpoints in the same experiment may have mismapping issues; care should be taken when interpreting results for these viewpoints. Despite providing the ability to generate 3C profiles with over 100,000 reporters, interpretable profiles can be generated from replicates with a few thousand reporters, as long as a high quality 3C library is used.

## Troubleshooting

### Troubleshooting advice can be found in Table 3.

## Figures and Tables

**Table 1:** 3C digestion efficiency qPCR primers.

Assay Set	Sequence	Site	*Dpn*II	*Nla*III
*Homo sapiens* (hg38)				
Hs 1 forward	5’-GTCAGAAATAACAGGAAACCCAAA-3’	chr22:46,257,116-46,257,137	Cut-site	Cut-site
Hs 1 reverse	5’-TTACTTGTCGAACCCAGAAGAC-3’	chr22:46,257,190-46,257,212		
Hs 2 forward	5’-GAGAATGGCCACATACAAGTAGA-3’	chr22:46,257,407-46,257,429	Fragment	Fragment
Hs 2 reverse	5’-GGAGTTGTCAACACAAGCATATC-3’	chr22:46,257,480-46,257,502		
***Mus musculus* (mm9)**				
Mm 1 forward	5’-GGAGAAAGAAGGCTGGTGTTAT-3’	chr15:85,650,603-85,650,624	Cut-site	Fragment
Mm 1 reverse	5’-TATCTGAGTTGGACAGCATTGG-3’	chr15:85,650,686-85,650,707		
Mm 2 forward	5’-TTATCTTGCATTTGCCAACTCG-3’	chr15:85,650,801-85,650,822	Fragment	Cut-site
Mm 2 reverse	5’-TGGGTTTCCCTGATTCTGAAA-3’	chr15:85,650,880-85,650,900		
***Drosophila melanogaster* (dm6)**				
Dm 1 forward	5’-CAGGCCAACACACATTGTATC-3’	chr3R:23,023,063-23,023,083	Cut-site	NA
Dm 1 reverse	5’-CGGCAGGCAAATCGAATAAA-3’	chr3R:23,023,146-23,023,165		
Dm 2 forward	5’-TGTTAGTCCCTGCCTCTGTA-3’	chr3R:23,023,278-23,023,297	Fragment	
Dm 2 reverse	5’-AAGTAACAGCAGCTGGAATAGG-3’	chr3R:23,023,358-23,023,379		

**Table 2:** Example calculations of digestion efficiency.

Sample^a^	Assay Set	Avg. CT	ΛCT (Cut-site − Fragment)	ΔΔCT (C1 − C2)	Digestion Efficiency^b^
**Control 1 (Undigested)**	Fragment	21.211	-0.168	-2.706	84.76%
	Cut-site	21.043			
**Control 2 (Un-ligated)**	Fragment	20.884	2.538		
	Cut-site	23.422			

a For low-input samples genomic DNA can be used instead of Control 1, and the 3C library in place of Control 2. Due to re-ligation a lower digestion efficiency is expected.

b Efficiency = 100 x (1-2^ΔΔCT^).

**Table 3:** Troubleshooting table.

Step	Problem	Cause	Solution
Reagent Setup	Excess Lysis buffer	Small number of samples.	To make smaller volumes of lysis buffer, one cOmplete Protease Inhibitor Cocktail tablet can be dissolved in 2 mL of PCR grade water to generate a 25× stock. This can be aliquoted and stored at - 20°C for several months.
9	Fewer than 5 x 10^6^ cells	Working with a rare cell population or limited number of cells following cell sort.	For fixation, PBS wash and lysis the volumes can be scaled down to accommodate fewer cells (down to 2 x 10^4^ cells). Maintain cells at ~1 x 10^6^ cells per 1 mL of growth media except for ≤1 x 10^6^ cells where 1 mL of media should be used and fixation and lysis performed in a 1.5 mL tube. Perform digestion reactions in 200 μL for between 2 x 10^4^ and 5 x 10^6^ cells.
	More than 5 x 10^6^ cells	Working with a cell line	For fixation, PBS wash and lysis the volumes can be scaled up to accommodate more cells. Maintain cells at ~1 x 10^6^ cells per 1 mL of growth media. For greater numbers of cells, perform multiple, parallel digestions and combine material in 300 μL of TE buffer after nuclear isolation.
37	Phenol use is not desireable or prohibited	Phenol is a dangerous chemical	Use of column extraction is possible and is considerably faster, e.g. Qiagen DNeasy Mini kit can used from the point where nuclei are pelleted, step 32. However, also pellet Control 1 and Control 2, then increase Proteinase K treatment to 4 hours at 65°C, and elute the DNA from the columns using the volumes outlined at step 45, before preceding to Quality Control.
46	DNA not visible in agarose gel	Low amount of DNA because of low-cell contraints	Run 1 μL of each sample on a Genomic DNA ScreenTape.
47	Low DNA yield	Loss of nuclear pellet	The nuclear pellet can be hard to see and may accidentally be disturbed. If suffering low DNA yields, retain the supernatant and perform Phenolchloroform isoamyl alcohol DNA extraction. A good Nuclear 3C library should have >90% of DNA within the nuclear pellet. The combined DNA from the nuclear pellet and the supernatant is equivalent to an *in situ* 3C library.
		Incomplete de-crosslinking	Perform decrosslinking overnight
		Incomplete precipitation	Freezing at -80°C overnight may be beneficial for DNA yield, particularly for low-input samples.
48	No control DNA	Working with low cell numbers	For low-input samples (≤150,000 cells), where very little DNA is available for controls, digestion efficiency can be directly calculated from re-ligated 3C libraries against a genomic DNA input control. Note that due to re-ligation into the original fragment configuration, lower values for digestion will be observed than for a true digestion control.
49	Low digestion efficiency	Short digestion period or sub-optimal enzyme activity	The total digest time should be 20-24 hours. Additional restriction enzyme can be added at each optimal enzyme activity of the three timepoints (steps 21-23) for cells generating low digestion efficiency.
	Non-exponential amplifiction	Reaction conditions for primers not optimized to thermocycler	Perform a dilution series analysis wigh genomic DNA and include a melt curve to ensure no primer dimers are being produced.
61	DNA not at correct size	Sonicator settings not optimized	Each sonicator may vary and should be set accordingly. Settings for sonication should be first determined by testing with high molecular weight genomic DNA rather than wasting 3C library. It is important to take into account the mass of DNA being sheared.
77	Vacuum centrifuge is not available	Specific equitment may not available	DNA may be purified by AMPure XP SPRI bead clean-up (e.g. steps 53-59) with elution into 40.2 μL of Universal Enhancing Oligonucleotides (6.7 μL per library).
86	Beads stick to plastic	High affinity of streptavidin beads for plastic tubes	Streptavidin dynabeads tend to stick to plastics. We find this effect is minimised by using high-quality, non-sticky tubes, from Sorenson BioScience (39640T).
116	Loss of DNA after capture	Failed PCR reaction, user error during DNA bead clean-up.	Captured material is amplified off the beads in two batches. Although these reactions can be performed simultaneously, it is prudent to do each individually to protect against error or misfortune and to ensure adequate amplification has occurred.
	Low DNA yield post capture	Incomplete hybridization	A longer hybridization time of 68-72 h may increase capture yield.
138	Beads stick to plastic	High affinity of streptavidin beads for plastic tubes	Streptavidin beads tend to stick to plastics. We find this effect is minimised by using high-quality, non-sticky tubes, from Sorenson BioScience (39640T).
159	Loss of DNA after capture	Failed PCR reaction, user error during DNA bead clean-up	Captured material is amplified off the beads in four PCR reactions (two per hybridisation reaction). Here, these reactions are performed simultaneously, though it is possible to do these in two batches to protect against error or misfortune and to determine if adequate amplification has occurred.
172	Tiled-C matrix not generated	Using coodinates for a single viewpoint not a region	Change the bed file coordinates to match the Tiled-C targeted region including the start of the first targeted fragment and the end of the and last targeted fragment.
	Interaction matrix not generated	Using Capture-C configuration settings	Set analysis method in config.xml to either “tiled” for Tiled-C or “tri” for Tri-C.

## Data Availability

Example results were generated by analysing GSE129378^10^ [https://www.ncbi.nlm.nih.gov/geo/query/acc.cgi?acc=GSE129378].

## Box 1 3C Quality Control

The success of Capture-C methods relies upon the generation of high quality 3C material, with a high degree of digestion. To ensure the quality of 3C material two controls are prepared (steps 17 and 25). Control 1 contains undigested material and ensures DNA was not degraded prior to digestion. Control 2 contains digested, but un-ligated chromatin. These two controls, along with the ligated 3C library are assessed both qualitatively, using gel electrophoresis, and quantitatively, using real-time PCR.

### Qualitative Analysis

To visually inspect 3C library and control DNA, separate using a 1% (wt/vol) agarose gel with a moderate speed, ~70 mA, or using a Genomic DNA ScreenTape in a TapeStation instrument (Fig. 2a). Because of the low DNA requirements, a TapeStation is preferable when working with low-input samples^9^. Control 1 should contain a single band of high-molecular weight DNA that is not degraded. Control 2 should contain a smear of low molecular weight fragments. Low digestion efficiency can be associated with a faint band of high molecular weight DNA. The ligated 3C library should have increased in molecular weight due to concatenation, and resemble Control 1. Although complete ligation will result in a greater number of informative junctions being formed, libraries with partial ligation can still be used for Capture-C. Where DNA is limiting, most commonly when working with low cell numbers, it is possible to asses both Controls and 3C material using a genomic screentape in a TapeStation, looking for a similar pattern of DNA distribution for each of the three samples. Qualitative assessment, using Tapestation profiles with small amounts of DNA can also be used to ensure indexing reactions proceed as expected (Fig. 2b).

### Quantitative Analysis

Real time PCR is performed with primers that amplify across a restriction cut site, Cut-site Primers, or within a restriction fragment, Fragment Primers (Fig. 3c, Table 1). Fragment Primers will amplify to the same extent in both Control 1 and Control 2, providing a loading control for quantitative PCR (Fig. 3c). The Cut-site Primers will readily amplify in Control 1, but due to digestion, have reduced amplification in Control 2. This difference in amplification allows the quantification of digestion efficiency (Table 2). We recommend that libraries have at least a 70% digestion efficiency for use. When working with low-input samples, it is possible to determine cutting efficiency using the re-ligated 3C library and a genomic DNA control, however this will result in a lower calculated digestion efficiency due to re-ligation of DNA into its original configuration.

## Box 2 Modifications for Increased Specificity

The high-resolution and depth of signal achieved by Capture-C is due to its ability to specifically sequence target fragments, resulting in high numbers of unique reporters per viewpoint^46^. Two additive protocol steps provide this high-specificity sequencing. The first adaptation, named double capture, uses repeated enrichment to achieve a 160-fold increase in target sequence over single capture, generating 30-50% on-target sequence (Fig. 4). The second adaptation uses titration of oligonucleotides to reduce non-specific enrichment, generating 30-40% on-target sequencing following a single capture^10^. When the two methods are combined, up to 98% of mapped read pairs contain the target fragment.

As the number of probes varies between capture designs, a specific probe concentration must be calculated for each capture. The optimal concentration for capture with a single oligonucleotide is ~2.9 nM. For a pool of oligonucleotides, the DNA concentration is simply scaled by the number of unique oligonucleotides.

 Required Pool Concentration =2.9nM× Number of Probes 

It is important to note that this value was determined using mammalian cells, when working with other organisms a rule of thumb adjustment may be appropriate. For example, the Drosophila genome is roughly 15 times smaller than the human genome, so a target fragment will be 15 times more common when capture is performed on the same mass of indexed 3C library. Scaling the amount of probe (increase concentration 15-fold) or DNA (decrease amount 15-fold) to reflect this will increase the efficiency of capture. However, it is important to note the higher amounts of DNA are likely to generate better results due to the increased complexity of the library.

Pooled probe stocks are generated by combing equimolar amounts of probes at 1 μM (or a value greater than the calculated Required Pool Concentration). The concentration of this pooled stock is 1 μM, which can then be diluted to the calculated Required Pool Concentration as needed.

Although double capture is essential for high-specificity sequencing when targeting dispersed elements (NuTi Capture-C, Tri-C) it provides little benefit to contiguous designs (Tiled-C). For Tiled-C, the combination of titrated probes and enrichment at both sides of interaction junctions provides highly specific sequencing; 80-90% on target following single capture^13^.

## Editorial Summary

Capture-C detects DNA regions in close proximity in the nucleus, relying on oligonucleotide probes to enrich fragments of interest. This protocol describes several variations of this modular approach and a computational pipeline.

## Code Availability

CapCruncher can be used following direct installation from Bioconda or accesed via GitHub^91^ (https://github.com/sims-lab/CapCruncher/releases).
